# Integrative analysis of reference epigenomes in 20 rice varieties

**DOI:** 10.1038/s41467-020-16457-5

**Published:** 2020-05-27

**Authors:** Lun Zhao, Liang Xie, Qing Zhang, Weizhi Ouyang, Li Deng, Pengpeng Guan, Meng Ma, Yue Li, Ying Zhang, Qin Xiao, Jingwen Zhang, Hongmeijuan Li, Shunyao Wang, Jiangwei Man, Zhilin Cao, Qinghua Zhang, Qifa Zhang, Guoliang Li, Xingwang Li

**Affiliations:** 1 National Key Laboratory of Crop Genetic Improvement, Huazhong Agricultural University, 1 Shizishan Street, Hongshan District, 430070 Wuhan, Hubei China; 2 Department of Resources and Environment, Henan University of Engineering, 1 Xianghe Road, Longhu Town, 451191 Zhengzhou, Henan China; 3 Agricultural Bioinformatics Key Laboratory of Hubei Province and Hubei Engineering Technology Research Center of Agricultural Big Data, Huazhong Agricultural University, 1 Shizishan Street, Hongshan District, 430070 Wuhan, Hubei China

**Keywords:** Agricultural genetics, Epigenomics, Plant genetics

## Abstract

Epigenomic modifications are instrumental for transcriptional regulation, but comprehensive reference epigenomes remain unexplored in rice. Here, we develop an enhanced chromatin immunoprecipitation (eChIP) approach for plants, and generate genome-wide profiling of five histone modifications and RNA polymerase II occupancy with it. By integrating chromatin accessibility, DNA methylation, and transcriptome datasets, we construct comprehensive epigenome landscapes across various tissues in 20 representative rice varieties. Approximately 81.8% of rice genomes are annotated with different epigenomic properties. Refinement of promoter regions using open chromatin and H3K4me3-marked regions provides insight into transcriptional regulation. We identify extensive enhancer-like promoters with potential enhancer function on transcriptional regulation through chromatin interactions. Active and repressive histone modifications and the predicted enhancers vary largely across tissues, whereas inactive chromatin states are relatively stable. Together, these datasets constitute a valuable resource for functional element annotation in rice and indicate the central role of epigenomic information in understanding transcriptional regulation.

## Abstract (web summary)

Comprehensive epigenomic maps of various rice varieties are still unavailable. Here, the authors report the development of eChIP as a fast and low-input upgrade of regular plant ChIP-seq protocol for epigenome analysis of 20 rice varieties and annotate over 80% of the genome with different epigenome properties for transcriptional regulation.

## Introduction

Rice (*Oryza sativa*) is one of the most important staple crops in the world and a model for plant genomic research. A major challenge in rice biology is understanding how conserved genome information translates and contributes to the formation of important agronomic traits and adaptation to various environmental challenges^1,2^. Chromatin states (CSs), which are determined mainly by a variety of epigenomic features, dictate genome activities and transcriptional regulation^3,4^. In addition, CSs are instrumental for understanding how genetic variants affect epigenetic information, which leads to variation in gene expression and traits. Epigenomic information, including DNA methylation, posttranslational histone modification, and chromatin accessibility, has been characterized in rice and other plants^5^. These studies provide insight into the epigenomic features of *cis*-regulatory elements and coordinative effects of active and inactive histone marks on transcriptional regulation^6–8^. Moreover, many studies have revealed the functions of genes encoding various chromatin modification factors^9–11^ and characterized genome-wide profiles of some histone marks and DNA methylation^12–15^. Despite these significant advancements, comprehensive epigenome maps across different tissues and distinct genetic backgrounds are still lacking for elucidating the dynamics of CSs and their effects on dynamic expression profiles and for assessing genetic variations affecting gene expression in plants. One of the major obstacles is the low efficiency of ChIP-Seq experiments in plants.

In this study, we develop an eChIP-Seq method and, together with other approaches, construct comprehensive epigenome maps in distinct rice tissues from Minghui 63 (MH63), Zhenshan 97 (ZS97), Nipponbare (Nip) and 17 other varieties. Our integrative analyses provide a broad overview of epigenomic landscapes, promoter chromatin features, enhancer predictions, epigenomic dynamics in different tissues, and effect of genetic variation on these rice varieties, as comprehensive rice functional DNA elements maps that lay the foundation for rice ENCODE project (The Encyclopedia of DNA Elements).

## Results

### Mapping epigenomic marks in 20 rice varieties

**Fig. 1:**
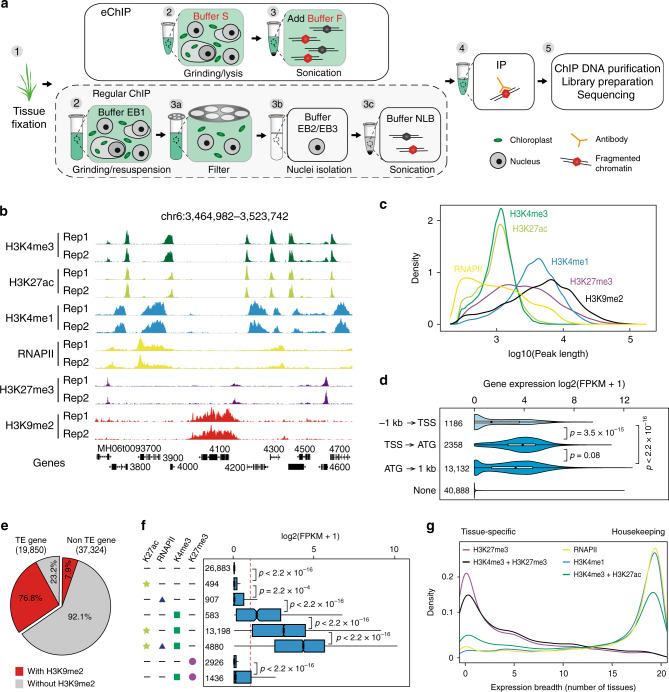
Histone modification landscapes profiled by eChIP-Seq in rice. **a** Schematic diagram of the eChIP and regular ChIP methods. Both methods start fixing tissues with formaldehyde, followed by grinding tissues to fine powder, homogenate lysis, chromatin sonication, IP (immunoprecipitation) with antibodies, ChIP DNA purification, library preparation, and sequencing. For eChIP, the lysed homogenate is directly sonicated for IP. In regular ChIP, the homogenate is first filtered through a mesh, and the isolated nuclei are then sonicated for IP. Steps 3a, 3b and 3c in regular ChIP are replaced by step 3 in eChIP. More details are shown in “Methods”. **b** Genome browser screenshot showing eChIP-Seq data for a young leaf of MH63. **c** Density distribution of the lengths of histone mark-modified and RNAPII-occupied regions in young leaf of rice. **d** Distribution of gene expression from the young leaf. The genes were divided into different categories based on the H3K4me3 peak positions relative to TSS and ATG of genes. TSS transcription start sites. Peak numbers of each categories are shown. **e** Distribution of TE genes and non-TE genes, marked with or without H3K9me2, in the young leaf of rice. **f** Expression levels of genes with promoters marked by different histone modifications and RNAPII. Numbers of genes in promoter categories are shown. Short line means that there is no certain histone modification or RNAPII occupancy. Boxplots in (**d**) and (**f**) include a median with quartiles and outliers above the top whisker. The statistical analysis was performed using two-side Wilcoxon test. The numbers indicate the sample size used in the analysis. **g** Breadth of expression (number of tissues that a gene is expressed in) of genes modified by different histone marks and RNAPII. Source Data underlying Fig. 1f, g are provided as a Source Data file.

To characterize chromatin epigenomic features and identify DNA regulatory elements in rice, we first developed an enhanced chromatin immunoprecipitation (eChIP) method for plants that highly improved the efficiency of chromatin extraction via direct sonication of formaldehyde-fixed tissues (Fig. 1a). Most (72.1%) fragmented chromatin could be recovered for immunoprecipitation (IP) by using eChIP (Supplementary Fig. 1a), compared to 4.1% obtained by the regular ChIP method^16,17^. The eChIP method reduced the required amount of starting material to 0.01 g for one IP with equal or better data quality, compared to the regular method using approximately 1–5 g of tissue^16–18^ (Supplementary Fig. 1 and Supplementary Table 1). Notably, chlorophyll DNA reads were minimal (~0.1%) in the eChIP-Seq dataset, although no specific step for chlorophyll removal was introduced (Supplementary Fig. 1h). Furthermore, eChIP also works well for modified histones and transcription factors in other monocotyledon and dicotyledon plants such as *Arabidopsis*, maize, and *Brassica napus* (Supplementary Fig. 2). These results demonstrated that eChIP is a fast (Supplementary Fig. 1i) and robust ChIP method in plants when only small amount of starting material is available.

By using the eChIP-Seq method, we characterized the genome-wide enriched regions of five histone modification marks (H3K4me3, H3K27ac, H3K4me1, H3K27me3, and H3K9me2) in four tissues (young leaf, mature leaf, root, and panicle) from MH63, ZS97 and Nip using validated antibodies (Supplementary Figs. 3, 4). We also generated the same ChIP-Seq datasets in young leaf of another 17 rice varieties representing a broad range of the global rice germplasm to examine the impact of genetic variations on epigenome profile (Supplementary Table 2). We further generated datasets for genome-wide DNA methylation, open chromatin regions, RNA polymerase II (RNAPII) binding sites, and the transcriptome for these tissues and varieties. Collectively, we generated 510 datasets for annotating the epigenomes of 20 rice varieties for downstream analyses (Supplementary Table 2).

Consistent with previous reports^12,14^, active histone marks were associated with active genes, with low DNA methylation levels in the 5′ and 3′ regions of gene bodies, and with high DNA methylation levels in the transcribed regions (Supplementary Fig. 5a, b). Histone marks H3K4me3 and H3K27ac frequently co-occurred close to the 5′ regions of active genes (Fig. 1b and Supplementary Fig. 5c) with narrow peaks (around 1 kb of width) (Fig. 1c). The majority (93%) of H3K4me3 peak summits were located downstream of transcription start sites (TSSs), and the transcription activity of these genes was significantly higher than that of genes with the summits located upstream of TSS (Fig. 1d). H3K4me1 marked the transcribed region of active genes (Supplementary Fig. 5c) with wider peaks (~6 kb) (Fig. 1c). Repressive H3K27me3 marks were enriched throughout the gene body, with slightly higher abundance near the TSS (Supplementary Fig. 5c), and covered a higher proportion of LINE (long interspersed nuclear element) and SINE (short interspersed nuclear element) retrotransposons and DNA transposons than did active marks (Supplementary Fig. 6b). Heterochromatin mark H3K9me2 was associated with *Gypsy* and *Copia* retrotransposons (Supplementary Fig. 6b), which accumulated mainly in the intergenic regions and exhibited significantly higher levels of DNA methylation than did other transposons (Supplementary Fig. 6a, c). Of the total TE genes, 77% were modified by H3K9me2 in different tissues (Fig. 1e and Supplementary Fig. 5d). In addition, genes with TEs inserted into exons harbored a larger proportion of H3K9me2 modifications and contained a larger proportion of unexpressed genes than did genes with TEs located in other regions (Supplementary Fig. 6d–f). This distribution revealed synergistic regulation of gene expression by TE insertion sites and H3K9me2 modification. Chromatin-accessible regions identified by formaldehyde-assisted isolation of regulatory elements followed by sequencing (FAIRE-Seq) were mainly located upstream of H3K4me3 peaks (Supplementary Fig. 5c). Surprisingly, RNAPII was more abundant in the vicinity of transcription terminal sites (TTSs) than in TSS regions (Supplementary Fig. 5c), indicating a potential role of TTS regions in transcriptional regulation.

To better understand the roles of H3K4me3 and H3K27ac modifications and RNAPII occupancy in transcriptional regulation, we examined the expression of genes harboring different combinations of histone marks (Fig. 1f). In contrast to the genes marked by H3K27ac or RNAPII with the low or even no expression, the genes marked only by H3K4me3 were relatively highly expressed. Moreover, once a gene had been marked by H3K4me3, its expression level increased significantly by a progressive addition of H3K27ac modification and RNAPII occupancy. Taken together, these observations suggested that H3K4me3 modification might be responsible for the initiation of gene transcription, whereas H3K27ac modification and RNAPII occupancy had an incremental effect on regulation of transcript abundance. This is similar to that in mammals^19^, indicating that the transcriptional regulation of these histone modifications on genes may be conserved in eukaryotes.

We also characterized the expression patterns of genes modified by various epigenetic marks using RNA sequencing (RNA-Seq) data from 20 representative rice tissues (Fig. 1g and Supplementary Table 3). Genes associated with RNAPII occupancy and active marks (H3K4me1, H3K4me3, and H3K27ac) tended to be expressed in the majority of examined tissues. Conversely, most genes with the repressive mark (H3K27me3) and bivalent marks (H3K4me3 and H3K27me3) tended to be expressed in a small number of tissues. These results indicated that highly expressed genes are more likely expressed constitutively as housekeeping genes, while H3K27me3-marked genes are more likely to be tissue-specifically expressed.

### Characterization of comprehensive epigenome maps in rice

**Fig. 2:**
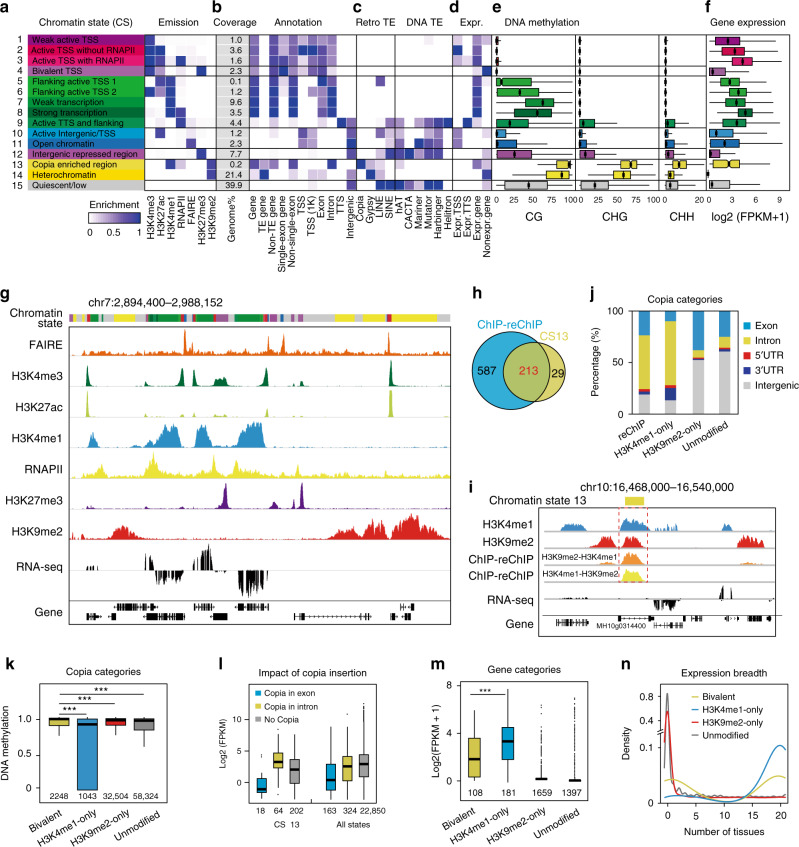
Comprehensive epigenome map in MH63 young leaf. **a** Chromatin state definitions, abbreviations, and composition (emission probability) of histone marks, open chromatin (FAIRE), and RNAPII occupancy. **b** Genome coverage and genomic annotation enrichments. TSS transcription start sites, TTS transcription terminal sites. **c** TE enrichments. Retrotransposons (retro TE; *Copia*, *Gypsy*, LINE and SINE) and DNA transposons (DNA TE; hAT, CACTA, Mariner, Mutator, Harbinger, and Helitron) are shown. **d** Active and inactive gene enrichments. **e** Methylation levels at CG, CHG, and CHH sites (H = A, C, or T). **f** Expression levels of genes associated with each chromatin state. Boxplots in (**e**) and (**f**) show the median, third and first quartiles. **g** Example of chromatin states. State colors are as in (**a**). **h** Venn diagram showing the overlap of H3K4me1-ChIP/H3K9me2-reChIP binding targets with chromatin state 13 regions. **i** Genome browser screenshot showing H3K4me1, H3K9me2 eChIP-Seq data and H3K9me2-ChIP/H3K4me1-reChIP-Seq, H3K4me1-ChIP/H3K9me2-reChIP-Seq data for a representative bivalent gene in chromatin state 13. **j** Distribution of different *Copia* categories in the indicated regions of the rice genome. Each type of sample number was indicated in (**m**). **k** DNA methylation levels of *Copia* in the indicated categories. The numbers indicate the sample size used in the analysis **l** Density plot of gene expression in chromatin state 13. Genes with *Copia* in an exon or an intron, and non-*Copia* genes are shown. Only gene with FPKM > 0.15 have been considered and the numbers indicate sample size used in the analysis. **m**, **n** Expression level and breadth of genes in the indicated categories. Boxplots in (**k**−**m**) include a median with quartiles and outliers above the top whisker. ***p < 0.001 from Wilcoxon ranked sum tests. Source Data underlying Fig. 2k−n are provided as a Source Data file.

We examined CSs based on the combinatorial patterns of different epigenetic marks. We first trained a 15-CS model using ChromHMM (v1.12)^3^ at 200-bp resolution along the genome for three tissues (young leaf, mature leaf, and panicle) from MH63 (Fig. 2a and Supplementary Fig. 7a, b). The rice genome was partitioned into four categories (active, repressive, inactive, and Quies) of CSs, with distinct levels of genome coverage, gene transcription, TE enrichment, and DNA methylation (Fig. 2a–g and Supplementary Fig. 7). The same CSs from different tissues and rice varieties showed similar epigenome properties (Supplementary Fig. 7).

The active states comprised active promoter-associated states (TssWk/CS1, TssA/CS2, TssAP/CS3), transcription-associated states (TssAFlnk1/CS5, TssAFlnk2/CS6, TxWk/CS7, Tx/CS8), an active TTS and flanking state (TtsAFlnk/CS9), and active intergenic states (IntA/CS10, OpenChr/CS11) (Fig. 2a–g and Supplementary Fig. 7). The active promoter-associated states had a higher frequency of H3K4me3 in common, as well as low enrichment of TEs with extremely low CG and non-CG methylation, which covered 8.3% of the genome. These states differed with respect to the presence and levels of other associated marks, primarily H3K27ac and RNAPII occupancy, leading to varying expression levels of their marked genes. The transcription-associated states were predominantly associated with H3K4me1 and accounted for 14.5% of the genome (Fig. 2a–g). The strong transcription state Tx was additionally marked with RNAPII occupancy, and the TssAFlnk1 and TssAFlnk2 states were marked with H3K27ac and H3K4me3. All transcribed states showed low enrichment of retrotransposons and DNA transposons, other than a moderate enrichment of retrotransposon LINEs. However, a higher CG methylation level in the core transcribed states than in the transition states (TssAFlnk and TtsAFlnk) was apparent, although extremely low CHG and CHH methylation was noted in all transcribed states. In addition, the differences in CG methylation level among these states were larger among highly expressed genes than among weakly expressed genes, leading to a more pronounced methylation profile (Supplementary Fig. 5b).

Notably, CS TtsAFlnk/CS9 was characterized by a high frequency of RNAPII occupancy and showed 45% enrichment for TTS and TTS flanking (Fig. 2a–g). Although similar states with higher RNAPII occupancy or H3K36me3 frequencies are observed in mammals^20,21^, no corresponding chromatin signature for the 3′ ends of genes in plants had been described. This state covered 3.6% of the genome, on average, with higher enrichment of DNA transposons than other active states and intermediate DNA methylation. Notably, Helitron, one kind of DNA transposons, was also significantly enriched in CS9, indicating its emergence from the transcription of gene 3′ ends.

Two intergenic active states were associated with higher frequencies of H3K27ac and FAIRE signal, respectively (Fig. 2a–g). These states included most of the candidate enhancer or other regions proximal to the expressed genes. They also showed relatively low DNA methylation and various enrichments for different TE types. CS IntA showed intermediate enrichment for LINE, and CS OpenChr was highly enriched for DNA transposons, including hAT, Mariner, Mutator and Harbinger.

The repressive states comprised TssBiv/CS4 and ReprPC/CS12 (Fig. 2a–g). In contrast to CS TssBiv marked by both H3K4me3 and H3K27me3 in the promoter region, CS ReprPC was predominantly marked by H3K27me3 alone and mainly enriched in large-scale intergenic regions. It covered 8.2% of the genome, with a higher level of LINE/SINE retrotransposons, DNA transposons, and intermediate DNA methylation than CS TssBiv.

The inactive states included two types of heterochromatin states (*Copia* and Het) (Fig. 2a–g). CS Het was defined by enrichment of H3K9me2 and covered the largest portion of the annotated region of the genome (20.1% on average). Most Het colocalized preferentially with intergenic regions (82%) and TEs (95%), comprising the majority (80%) of TE genes and a large proportion of the two largest classes of retrotransposons, *Gypsy* (56%) and *Copia* (36%). This state showed the highest DNA methylation, and the majority (97%) of genes covered with this state were not expressed. CS *Copia*, which was defined by the active mark H3K4me1 and inactive mark H3K9me2 (Fig. 2a), was a bivalent-like state unrecognized in previous reports^6,20^. We validated H3K4me1-H3K9me2 bivalent chromatin state by ChIP-re-ChIP-Seq (Fig. 2h, i), which covered a small proportion of the genome (0.2%) (Fig. 2b) and exhibited specific epigenomic properties. It showed the highest enrichment of the retrotransposon *Copia* (Fig. 2c). Compared with other *Copia* categories (H3K9me2-only and unmodified), bivalent *Copia* was frequently located within gene body regions, especially in introns, and showed the highest DNA methylation level (Fig. 2j, k). In addition, genes with *Copia* inserted into exons contained a larger proportion of unexpressed genes than did genes with TEs located in introns in the bivalent state (Fig. 2l). Compared with the H3K4me1-only category genes, the genes with bivalent states exhibited an intermediate and lower expression level and were placed in both tissue-specific and housekeeping expression categories (Fig. 2m, n).

The Quies state (CS15) was devoid of any modification but showed high enrichment of LINE/SINE retrotransposons and DNA transposons (Fig. 2 and Supplementary Fig. 7).

Taken together, these data revealed distinct genomic region profiles with different biochemical properties and highlighted the complex relationship between DNA methylation, TE enrichment, and gene transcription in various CSs. Except for the Quies state, approximately 81.8% of the rice genomes were annotated with at least one CS.

### Refinement of rice promoter with chromatin signature

**Fig. 3:**
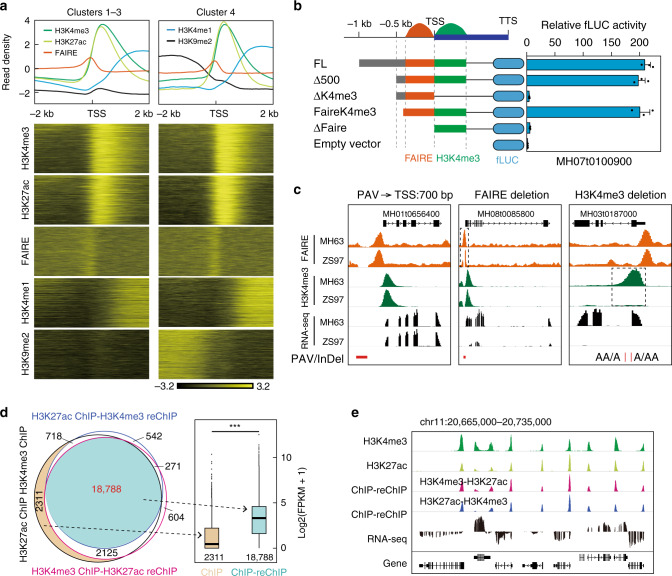
Epigenetic features of rice promoters and associated transcription patterns. **a** Comparison of promoter Clusters 1−3 and Cluster 4, defined by epigenetic modification patterns around promoters in Supplementary Fig. 7. **b** The refinement of promoter regions with H3K4me3 modification and open chromatin signal on transcription activation. A transient reporter assay was used to examine the effects of different promoter regions on gene expression in rice protoplasts. Left, schematic representation of fLUC reporter constructs fused with the full-length promoter (FL) and deletion variants (Δ500, ΔK4me3, FaireK4me3, and ΔFaire). Each value represents the mean ± standard error of mean (*n* = 3 biological replicates). Empty vector served as the negative control. Gene MH07g0100900 serves as a representative example. **c** Representative examples for transcription effects of promoter length, open chromatin, and H3K4me3-modified regions on transcription activity, validated by the presence of InDels and PAVs between MH63 and ZS97. Dotted boxes represent the regions with deleted peaks of FAIRE or H3K4me3 in ZS97. **d** Validation of co-occupation for H3K4me3 and H3K27ac using ChIP-reChIP. Venn diagram (left panel) showing the overlap of H3K4me3 and H3K27ac co-occupation sites dedicated using ChIP-reChIP-Seq and ChIP-Seq, respectively. Boxplots (right panel) for expression levels of the indicated gene category. Boxplots show the median, third and first quartiles. ***p < 0.001 from Wilcoxon test. The numbers indicate the sample size used in the analysis. **e** Genome browser screenshot showing H3K4me3- and H3K27ac-commodified genes. Source Data underlying Fig. 3b, d are provided as a Source Data file.

We next explored chromatin features in the vicinity of gene promoters by examining the profiles of different epigenomic marks along ±2 kb regions around TSS. We classified them into 15 clusters based on histone modifications and chromatin-accessible patterns (Supplementary Figs. 8–10). We divided these 15 promoter clusters into four distinct groups according to gene expression levels in four tissues and varieties (Supplementary Figs. 8–10). Interestingly, similar to clusters 1–3, genes in cluster 4 were highly enriched for active epigenomic marks around TSS, with an especially high enrichment of H3K9me2 beyond 500 bp upstream of TSS (Fig. 3a). This result implied that, instead of the H3K9me2-modified region, open chromatin and H3K4me3-modified regions were critical for transcription activation.

A transient reporter assay was performed to validate the roles of the H3K4me3-modified regions and open chromatin regions in transcription activity, as indicated by a firefly luciferase (fLUC) reporter under the control of truncated promoters in rice protoplasts (Fig. 3b and Supplementary Fig. 11). The majority (85%, 11/13) of the promoters containing both an H3K4me3-modified region and an open chromatin region (named FAIREK4me3) or regions further upstream of TSS (FL and Δ500) showed similarly high fLUC activity, while promoters without the FAIRE element (ΔFAIRE) or H3K4me3-modified region (ΔK4me3) displayed almost no or very low fLUC activity (Fig. 3b and Supplementary Fig. 11). This result indicated that the H3K4me3-modified region was crucial for transcriptional activity and that the open chromatin region (or only ~500 bp upstream of TSS) combined with the H3K4me3-modified region was essential for transcriptional activation of their genes.

To confirm these results, we used the naturally occurring genomic presence/absence variations (PAVs) and/or insertion/deletions (InDels) in MH63, ZS97, and Nip genomes overlapping with the identified FAIRE element and H3K4me3 modification to evaluate their effects on gene expression (Fig. 3c). The majority (92%) of PAVs located 500–1000 bp upstream of TSS did not affect the transcriptional activity of their closest genes. Six PAVs that reduced the enrichment of FAIRE peaks exhibited a corresponding reduction of transcriptional activity of their closest genes. For example, the FAIRE element upstream of the TSS of the MH08t0085800 gene was largely deleted in ZS97, resulting in a pronounced reduction of transcriptional activity in ZS97 compared with that in MH63 (Fig. 3c). We also identified 32 InDels that affected the enrichment of H3K4me3 peaks and exhibited largely reduced transcriptional activity in ZS97 (Fig. 3c). These findings strengthened the notion that both the FAIRE element and H3K4me3 modification are crucial for the transcriptional activity of a gene.

Since H3K4me3 and H3K27ac were frequently co-located in promoters, we generated H3K4me3-ChIP-H3K27ac-reChIP-Seq and H3K27ac-ChIP-H3K4me3-reChIP-Seq data to investigate the co-occupancy of H3K4me3 and H3K27ac in the same nucleosome (Fig. 3d, e). Of the total (23,224) binding sites with deduced overlapping from H3K4me3 ChIP-Seq and H3K27ac ChIP-Seq data, approximate 81% were verified through ChIP-reChIP method. These H3K4me3- and H3K27ac-comodified genes showed higher expression level than those genes modified by either H3K4me3 or H3K27ac only (Fig. 3d), suggesting H3K4me3 and H3K27ac have superposition effect in regulating gene expression.

### Dynamic epigenome landscapes across rice tissues

**Fig. 4:**
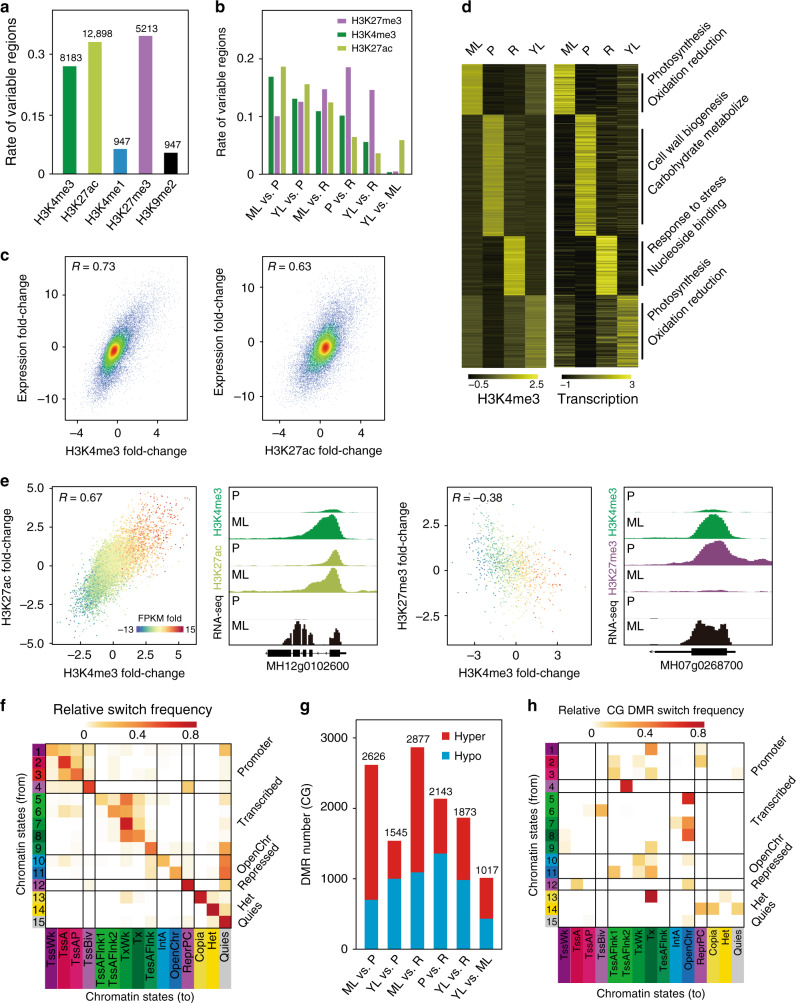
Dynamics of epigenome features and gene expression in MH63 tissues. **a** Number and rate of histone marks that were variable across four tissues (young leaf, mature leaf, panicle, and root). **b** The rate of variable histone marks between the indicated tissues. YL young leaf, ML mature leaf, P panicle and R root. **c** Correlations of log2-fold-change in single histone mark (*x*-axis) and gene expression (*y*-axis) between mature leaf and panicle. **d** Dynamics of H3K4me3 modification and transcription in four tissues. Heatmaps of tissue-specific H3K4me3 and the expression levels of genes. Biological GO functions enriched for differentially regulated genes are shown. **e** Correlations of log2 fold-changes of H3K4me3 and H3K27ac (left panel) or H3K27me3 (right panel) between panicle and mature leaf. Color scale indicates the fold-changes of gene expression. Each dot represents the degree of change between two histones and expression levels on the same gene. Only genes with both H3K4me3 and H3K27ac (or H3K27me3) marks are plotted here. Representative examples are shown. **f** Log10 ratio of the observed probability that a region switches from one chromatin state (row) to another (column). **g** Number of CG differentially DMRs in different tissues. The numbers indicate the sample size used in the analysis. **h** Log10 ratio of relative CG DMR switching frequency with which a region switches from one chromatin state (row) to another (column). Source Data underlying Figs. 4c−f, h are provided as a Source Data file.

We then systematically characterized the variability of chromatin marks in tissues. We observed greater dynamic changes in the active histone marks H3K4me3 and H3K27ac and the repressive mark H3K27me3 than those in the transcribed mark H3K4me1 and heterochromatin mark H3K9me2 (Fig. 4a). Notably, we found high variability in the H3K4me3, H3K27ac and H3K27me3 marks in heterogeneous tissues (such as young leaf vs. panicle, panicle vs. root), in contrast with the high variability in H3K27ac in homogeneous tissues (young leaf vs. mature leaf) (Fig. 4b). This result suggested relative stability of epigenomic landscapes of transcribed and heterochromatin regions compared with the higher variability at promoters in rice tissues, and H3K27ac might be responsible for chromatin variability at promoters between homogeneous tissues.

Next, we investigated the correlation between epigenetic marks and gene expression in heterogeneous tissues. The levels of H3K4me3 changes and H3K27ac changes are strongly positively correlated (*R* = 0.67) with gene expression changes, whereas the H3K27me3 changes showed a weak negative correlation (*R* = −0.38) with gene expression changes (Fig. 4e and Supplementary Figs. 12a and 13a). For example, most (64%) genes with higher H3K4me3 modification in mature leaves than in roots resulted in higher expression in mature leaves, as did the H3K27ac modification. Whereas 48% of genes with higher H3K27me3 modification in mature leaves than in roots exhibited lower expression in the mature leaves than in the roots. Based on gene ontology (GO) analysis, genes that exhibited tissue-specific enrichment in epigenetic marks were involved in different biological processes (Fig. 4d and Supplementary Fig. 12b). For example, genes that were differentially modified by H3K4me3 in mature leaves and were also enriched in mature leaves at the transcript level were enriched in the categories of photosynthesis and oxidative reduction processes. In addition, we observed a strong positive correlation in fold-changes between the H3K4me3 and H3K27ac; most (83%) genes showed changes in both H3K4me3 and H3K27ac also exhibited changes in gene expression (Fig. 4e and Supplementary Fig. 13b). In contrast, we observed a weak negative correlation between the bivalent mark (H3K4me3 and H3K27me3) changes in heterogeneous tissues (Fig. 4e and Supplementary Fig. 13b). In summary, similar to the study of other plants^15^, the H3K4me3, H3K27ac, and H3K27me3 dynamics were generally consistent with gene expression in rice tissues.

We also investigated the relative frequency of switches from one CS to another CS across different tissues (Fig. 4f). We found high switch frequency within promoter regions and transcribed regions, except for the relatively low-switch frequency from the weak transcription state defined by H3K4me1 to other states. This is consistent with the low variability in H3K4me1 (Fig. 4a). High switch frequencies were also apparent for the switch from CSs IntA and OpenChr to CS Quies, which could explain the robust dynamics of enhancers in different tissues. By contrast, low-switch frequency was notable in intergenic repressive, heterochromatin, and Quies states. This agrees with the high variability in active and repressive marks and the low variability in inactive mark across tissues (Fig. 4a). Interestingly, switching among the core promoter, transcribed, repressive, heterochromatin, and Quies CSs was rare, highlighting that these represent distinct types of regulatory elements.

We observed higher DNA methylation levels in root than in other tissues examined (Supplementary Fig. 12c), and the differentially methylated regions (DMRs) were more apparent in heterogeneous tissues than in homogeneous tissues (Fig. 4g and Supplementary Fig. 12d, e). Further, CG DMRs were enriched mainly in CSs that switched from active promoter regions to transcribed regions, between transcribed states and intergenic active-CSs, among inactive states, and from *Copia* to highly transcribed states (Fig. 4h). We observed the highest enrichment of non-CG DMRs between *Copia* and highly transcribed states (Supplementary Fig. 12f).

### Mapping rice *cis*-regulatory elements with enhancer activity

**Fig. 5:**
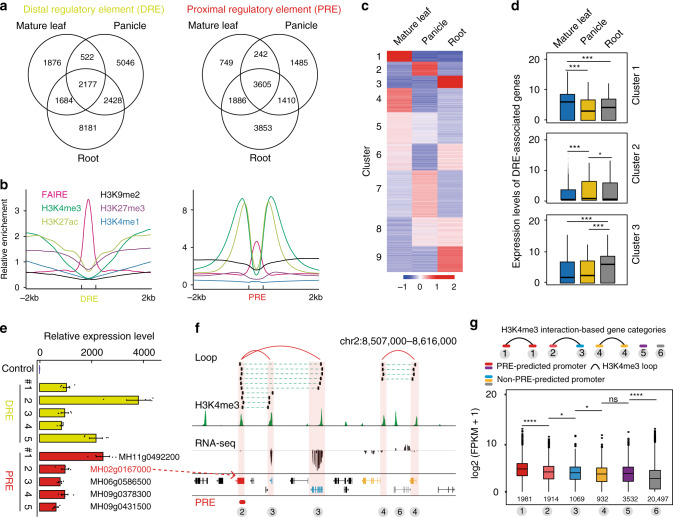
Epigenetic characteristics of open chromatin region and enhancer-like promoter. **a** Venn diagram showing the overlap between distal regulatory element (DRE) and proximal regulatory element (PRE) in the mature leaf, panicle, and root of MH63. DREs and PREs were defined in Supplementary Fig. 15. **b** Epigenetic features of DREs and PREs in mature leaf. Element regions were scaled to equal size. **c** Heatmap of tissue-specific DREs showing dynamics between the indicated tissues. Tissue-specific DREs were divided into nine clusters based on their signal intensities. **d** Boxplots of the expression levels of genes proximal to the tissue-specific DRE in the clusters defined in (**c**). *n* = 4113, 3067 and 3465 are the number of genes in clusters 1−3, respectively. ***p < 0.001 from Wilcoxon test. **e** Validation of DRE and PRE-predicted enhancers. Enhancer-like promoter refers to a promoter occupied by PRE. A transient reporter assay was used to examine the effects of these two types of regulatory elements on gene expression in rice protoplasts. One genic fragment (chr2:28,412,151-28,413,151) without epigenetic signal served as negative control. Each value represents the mean ± standard error of mean (*n* = 4 biological replicates). **f** A representative example for enhancer-like promoter with chromatin loops. The representative enhancer-like promoter (MH02g0167000, red) was validated in (**h**). **g** Expression level of genes in different categories based on H3K4me3-associated chromatin interaction models and PRE-occupancy. The number of different types of genes were labeled. Boxplots in (**d**) and (**g**) include a median with quartiles and outliers above the top whisker. ****p < 0.0001 and *p < 0.1 from Wilcoxon test; ns means no significant. The numbers indicate the sample sizes used in the analysis. Source Data underlying Fig. 5c−e, g are provided as a Source Data file.

We attempted to identify nonpromoter *cis*-regulatory elements using FAIRE-Seq-defined open chromatin regions following reported methods^22,23^, and examined their regulatory activity on gene expression. Based on the distribution and distance between FAIRE and TSS (Supplementary Fig. 14a), FAIRE regions were classified into distal regulatory element (DRE) and proximal regulatory element (PRE) (Supplementary Fig. 14b). Out of 21,914 DREs and 14,640 PREs, only small proportions (10% for DREs and 25% for PREs) were conserved across various tissues, suggesting appreciable dynamic changes of *cis*-regulatory elements during development (Fig. 5a). These enhancer candidates exhibited low CG and non-CG methylation, similar to active promoters (Fig. 5b). Moreover, the PRE-regions showed high levels of active marks H3K4me3 and H3K27ac, but with low levels of H3K27me3 and H3K9me2. Conversely, the active histone marks were low around DRE-regions (Fig. 5b), probably because they were located in the intergenic region. We further divided DREs into nine clusters (Fig. 5c) and found that the expression levels of most genes that flanked DREs in specific tissues were significantly higher than those in other tissues (Fig. 5d and Supplementary Fig. 14c, d). We also validated five randomly selected DREs by performing transient reporter assay using the STARR-Seq^24,25^ plasmid vector, and found that all of them have enhancer activity (Fig. 5e). These findings supported the appreciable dynamics of DREs across tissues and the impact of tissue-specific DREs on tissue-specific expression of the proximal genes.

Given that the active promoters involved chromatin loops could potentially capture putative enhancers^26^, we applied rice H3K4me3 ChIA-PET data^27^ to examine the regulatory function of PREs. We first validated five randomly selected PREs involved in chromatin interactions, and found that all of them showed enhancer activity (Fig. 5e, f). We thus speculated that, likes DREs, some PREs (or promoters) may have enhancer functions through chromatin looping in rice, similar to our previous report in mammalian cells^21^. Therefore, we named a promoter overlapping with a PRE-region showing enhancer activity as enhancer-like promoter. To comprehensively characterize those enhancer-like promoters, we performed integrative analysis of the rice seedlings H3K4me3 ChIA-PET data with PREs. The genes were divided into six categories based on H3K4me3-associated promoter−promoter interaction model and enhancer occupancy (Fig. 5g). We observed that the expression level of the genes in the enhancer-like promoter−promoter model was higher than those of genes in the promoter−promoter model (gene category 3 vs. 4). The genes with enhancer-like promoters and involved in chromatin interactions showed significantly higher expression levels than those not involved in interactions (categories 1, 2 vs. 5) (Fig. 5g). These results further support the existence of enhancer-like promoters, which are involved in chromatin interactions in the rice genome and play roles in transcriptional regulation.

### Genetic variants alter epigenetic marks and transcription

**Fig. 6:**
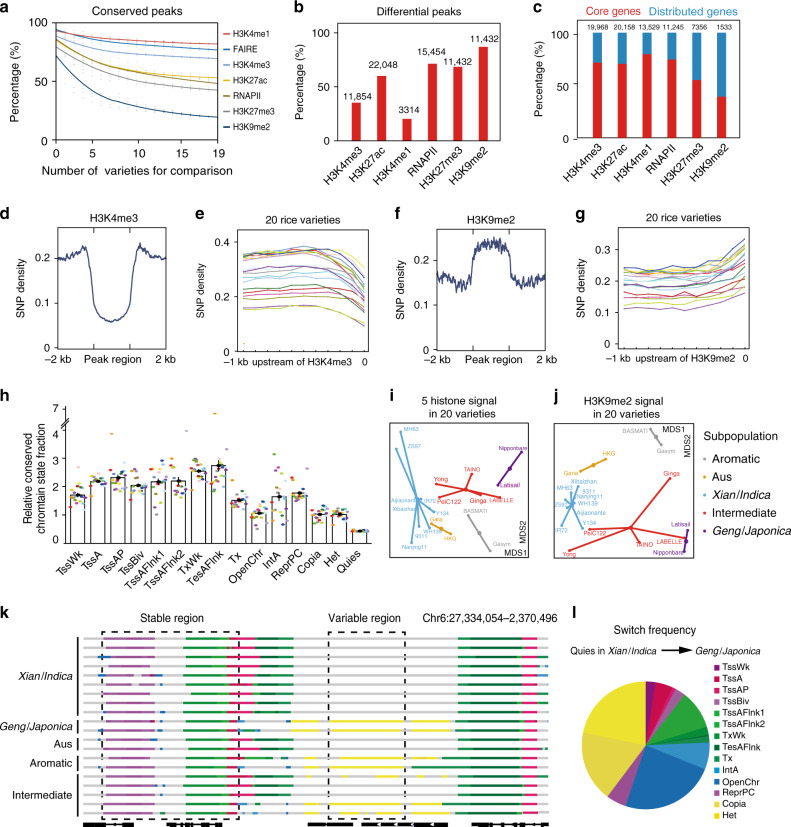
Correlations of genetic variations and epigenome properties in 20 rice varieties. **a** Fraction of regions for each mark signal with significant differences based on pairwise comparison. Differential regions were identified using DESeq2 (using a *q* value cutoff of 10^−2^ and a fold-change between individuals >1.5). **b** Number and percentage of histone marks that were differential in 20 rice varieties. **c** Distribution of core and distributed genes marked by different histone modifications in MH63. Core genes, present in more than 99% of 453 rice accessions; distributed genes, present in less than 99% of 453 rice accessions. The numbers in (**b**) and (**c**) indicate the sample sizes used in the analysis. **d**, **f** SNP (blue) density in H3K4me3 (**d**) and H3K9me2 (**f**) peak regions. **e**, **g** SNP enrichments upstream of H3K4me3-modified (**e**) and H3K9me2-modified (**g**) regions indicated by dotted boxes in 20 rice varieties. **h** The relative proportion of conserved chromatin state in the whole genome. **i**, **j** Nonmetric Multidimensional scaling (NMDS) plot of 20 rice varieties indicates their relationships based on similarity of five histone modification signal (**i**) and H3K9me2 signal (**j**). First two dimensions are shown as MDS1 versus MDS2. The five different subpopulations^52^ *Xian*/*Indica*, Aus, *Geng*/*Japonica*, aromatic and an intermediate group, are shown in different colors. **k** An example of a nonvariable and a variable region for chromatin state in 20 varieties. All epigenome datasets were mapped to the Nipponbare reference sequence MSU7.0^53^. State colors are as in (**g**). **l** The pie chart of the relative frequency of CS from *Xian*/*Indica* to *Geng*/*Japonica*. Source Data underlying Fig. 6a, h–j, l are provided as a Source Data file.

To evaluate the effect of genetic variation on histone modifications and gene expression, we analyzed the epigenome datasets from 20 rice varieties with abundant genetic variation (MH63, ZS97, Nip and 17 other varieties, see details in Supplementary Table 2). We found that, as the number of varieties increased, the proportion of differential epigenome signals gradually decreased (Fig. 6a) and the accumulation scores of all signals of histone marks and RNAP II occupancy tend to remain unchanged, indicating well representation of epigenome diversity of rice from 20 varieties (Fig. 6a). In addition, the accumulation score of H3K9me2 was the lowest, while the accumulation score of H3K4me1 signal was the highest (Fig. 6a). We also examined the variation of five histone marks and RNAPII occupancy across 20 varieties and found the dynamic of the heterochromatin-associated mark H3K9me2 was greater than that of other histone modification marks. H3K27me3 and RNAPII occupancy also showed high levels of variations, while H3K4me1 showed the lowest level of variation in 20 varieties (Fig. 6b), which suggest that heterochromatin-associated regions play a very important role in divergence of rice varieties.

Previous rice pan genome analyses have defined core genes (including candidate genes, present >99% of 453 rice accessions) and distributed genes (present <99% of 453 rice accessions)^28^. We examined epigenome features of these two categories of genes and found that the majority (72–80%) of active genes were classified in the core gene category, while the proportion of repressive and heterochromatin-associated genes were higher in the distributed gene category (Fig. 6c). This suggested that core genes tended to be active and constitutively expressed, whereas distributed genes were more likely to be specifically expressed or silent. We investigate the relationship between genetic variation and histone modifications in 20 rice varieties. Although the density of SNPs of each variety is different, they have similar distribution trends around the same histone modification region (Fig. 6e, g). Compared with the flanking regions of the peaks, the SNP density was least abundant in the H3K4me3 peak regions (Fig. 6d, e). However, the SNPs density in flanking regions of H3K9me2 peaks showed a significant upward trend in 20 varieties (Fig. 6g). These results indicate that active histone-marked regions and heterochromatin have differential capacity of bearing single nucleotide variants, which may have arisen as a consequence of functional selection.

To address the relationship between genetic variations and CSs, we found that the relative genome coverage and DNA methylation level among 20 varieties were relative stable (Supplementary Fig. 15a, b). We also found that CS7 (TxWk) and CS8 (Tx), which is mainly composed of H3K4me1 mark, is the most conservative CS, while CS10 (OpenChr), CS13 (*Copia*), CS14 (Het) and CS15 (Quies) were the least conservative ones across 20 varieties (Fig. 6h). By investigating the dynamics of CS transition between *Xian*/*Indica* and *Geng*/*Japonica* subgroups, we revealed that high-frequency CS state switches occurred from CS15 (Quies) to CS11 (OpenChr), CS13 (*Copia*) and CS14 (Het) (Fig. 6i and Supplementary Fig. 15c). In addition, we found the overall CSs of two varieties in the Aus group were more similar to *Xian*/*Indica*. Interestingly, some varieties in the Intermediate (Int) group tend to be close to *Xian*/*Indica* or *Geng*/*Japonica*, respectively (Fig. 6k and Supplementary Fig. 15d). We also measured the epigenome distance of 20 varieties and found the first two dimensions of variation for each of histone mark separated major groups of 20 rice varieties in nonmetric multidimensional scaling (NMDS) analysis (Fig. 6i, j and Supplementary Fig. 16a–f). We further found that the Intermediate group varieties LBELLE, Ginga and TAINO 38 were more similar to *Geng*/*Japonica* group, while Yong Chal Byo and PeiC122 were closer to the *Xian*/*Indica* group (Fig. 6i, j). Taken together, we observed a wide range of epigenetic differences among rice varieties, and these differences may be important resources for variety differentiation and adaptation to the environment in rice.

## Discussion

The plant epigenome research falls behind of mammalians’ and one of reason is that there is no efficient ChIP-Seq method for plants. Here, we developed an efficient ChIP-Seq method eChIP, which provides a platform for the rapid generation of a large amount of data for epigenome research and works well for all the examined plant tissues and species (including *Arabidopsis thaliana*, maize and *Brassica napus*). With eChIP method, we here present the most comprehensive epigenome maps across four tissues in three rice varieties (ZS97, MH63 and Nip) for which a high-quality reference genome sequence is available and another 17 representative core rice varieties. The rice ENCODE project will be valuable for elucidating the regulatory effects of DNA elements on transcription, growth and development. Similar projects for human^19^ and other model organisms^29–32^, the Roadmap Epigenomics Program^20,33^ and other projects have been implemented to comprehensively annotate functional genomic elements and generate reference epigenomic maps of cells and tissues of interest. One key to achieving these maps is that we developed a highly efficient and low-input eChIP method. With this method, we generated the largest epigenetic resource for rice to gain insight into the epigenomic landscapes, dynamics across tissues and regulatory functions as well as impacts of genetic variants on epigenome. With 510 libraries, we annotated nearly 81.8% of the rice genomes, including active DNA regulatory elements. Promoters-associated active and repressive marks are more dynamic than heterochromatin-associated inactive mark H3K9me2 across tissues. Genomic regions with specific combinations of epigenetic marks possess similar genome properties (such as genomic annotation and TE enrichments), DNA methylation levels, and transcriptional potential in distinct rice tissues and varieties, suggesting that epigenomic annotation has a powerful predictive function for genome activity.

Although promoters are widely used in the study of plant genetics, their precise lengths and chromatin features are not well defined. In many studies, the 2-kb region upstream of a gene TSS is considered arbitrarily as the promoter region of this gene. In this study, we refined the promoter regions for genes (at least for 1965 genes in cluster 4) in rice with FAIRE and H3K4me3 and validated the results with the LUC reporter assay. Remarkably, regions modified with H3K4me3, with the summits located predominantly (93%) downstream of TSS, are also essential for gene expression, suggesting that the transcription region modified by H3K4me3 serves as a critical component of rice promoters. Thus, the significance of the overlapping region between active promoter and transcription marked by H3K4me3 and the underpinning regulatory mechanism of gene transcription require further investigation, even though H3K4me3 is a well-established promoter feature.

We further identified extensive enhancer-like promoters that have function of transcriptional regulation through chromatin interactions in rice (Fig. 5g–i). This finding reveals the function of promoter in chromatin interaction, which is that promoter can not only regulate the expression of adjacent genes, but also regulate the expression of distant genes by chromatin folding. We also demonstrate the usefulness of the resulting epigenetic annotations for interpreting rice genetic variation. In a comparison among 20 rice varieties, we find that differences often occur in heterochromatin regions, providing insights on the genetic and evolutionary research.

Overall, our epigenome datasets, regulatory annotations, and integrative analyses have provided the most comprehensive maps of the rice epigenomic landscape. We expect that the eChIP method and the epigenomic map will be widely used by scientific communities for studies on genome interpretation, gene regulation, tissue development, crop genetic improvement, and epigenomic annotations of other plants.

## Methods

### Plant material and tissue collection

Three rice varieties, MH63 and ZS97 (the parents of one of the most widely cultivated rice hybrid, Shanyou 63, in China), and Nip, were used. The four tissues analyzed in the current study were the root, young leaf, mature leaf, and young panicle. Young leaves of other 17 rice varieties (Supplementary Table 2) and *OsbZIP23*-overexpressing plants^34^ were also collected. Germinating seeds were obtained by soaking dry seeds in water for 72 h at 37 °C. For roots and young leaves, the germinated seeds were grown in a phytotron with the day/night cycle set at 14 h/10 h and a temperature of 32 °C/28 °C. Roots were obtained 7 d after planting on moist filter paper, and young leaves were obtained from 2-week-old plants cultured hydroponically as described^35^. Approximately 20-d-old seedlings were transplanted to the field and managed under normal agricultural conditions on the experimental farm of Huazhong Agricultural University, Wuhan, China. Young panicles and mature leaves at the floral induction stage were harvested. Young leaves of 2-week-old *Arabidopsis* (Col-0), *Brassica napus* (B409), and maize (B73) after germinating were harvested for eChIP experiments.

### Antibody specificity validation

Western blot and dot blot experiments were used to confirm the specificity of modified histones. Nuclear lysate was extracted from MH63 plants. Western blot was performed as reported^36^. Proteins were separated by Sodium dodecyl sulfate-Polyacrylamide gel electrophoresis (SDS-PAGE), blotted, and probed with antibodies (1:1000 dilution) against modified histone according to standard procedures. For dot blot, the indicated polypeptides on nitrocellulose membranes were blocked with 3% bovine serum albumin (BSA) and incubated with the indicated primary antibody (1:1000 dilution) and secondary antibody (1:5000 dilution). After incubating with enhanced chemiluminescence (ECL) reagent (Pierce, 32016), membranes were exposed according to standard procedures.

### eChIP-Seq library preparation

Tissues were crosslinked with 1% formaldehyde for 10–15 min and quenched with 0.2 M glycine at room temperature. Approximately 0.1 g or the indicated amounts of samples were used for each eChIP-Seq assay. Samples were ground in liquid nitrogen into fine powder and then lysed in 300 μl of Buffer S (50 mM HEPES-KOH (pH7.5), 150 mM NaCl, 1 mM Ethylene Diamine Tetraacetic Acid (EDTA), 1% Triton X-100, 0.1% sodium deoxycholate, 1% SDS) for 10 min at 4 °C. The homogenate, containing the entire chromatin from the ground sample, was mixed with 1.2 ml of Buffer F (50 mM HEPES-KOH (pH7.5), 150 mM NaCl, 1 mM EDTA, 1% Triton X-100, 0.1% sodium deoxycholate), and the chromatin was fragmented into 200–600 bp by sonication using a Bioruptor (Diagenode). The lysates were centrifuged at 20,000 × *g* for 10 min at 4 °C, and the supernatant (containing most of the fragmented chromatin) was transferred to a new tube for ChIP. For subsequent analysis, 20 μl of supernatant was used as the input sample. ChIP was performed using antibodies against the following: H3K4me1 (ABclonal, A2355), H3K4me3 (Millipore, 07-473; ABclonal, A2357), H3K9me2 (Abcam, ab1220), H3K27me3 (ABclonal, A2363), H3K27ac (ABclonal, A7253), and RNAPII (BioLegend, 920102). In doing so, 50 μl of Dynabeads® protein G beads (Life Technologies, 10003D) were washed with 300 μl of PBST buffer (phosphate-buffered saline (PBS) with 0.1% Tween 20) twice and resuspended in 200 μl of PBST buffer. Antibody solution (5–10 μl) was added to the beads and incubated for 1–6 h on a rotator at 4 °C to form antibody-bead complexes. The antibody-bead complexes were washed with PBST twice and incubated with precleared chromatin for 8 h or overnight at 4 °C on a rotator to immunoprecipitate the target chromatin. The immunoprecipitated chromatin was washed subsequently with low-salt ChIP buffer (50 mM HEPES-KOH, 150 mM NaCl, 1 mM EDTA, 1% Triton X-100, 0.1% sodium deoxycholate, 0.1% SDS) (three times), high-salt ChIP buffer (low-salt ChIP buffer replaced 150 mM NaCl with 350 mM NaCl) (twice), ChIP wash buffer (10 mM Tris-HCl pH 8.0, 250 mM LiCl, 0.5% NP-40, 1 mM EDTA, 0.1% sodium deoxycholate) (once), and TE buffer (10 mM Tris-HCl, pH 8.0, and 1 mM EDTA) (twice). The protein-DNA complexes were eluted from beads by adding 100 μl of freshly prepared ChIP Elution buffer (50 mM Tris-HCl pH 7.5, 10 mM EDTA, 1% SDS) for 15 min at 65 °C with agitation at 900 rpm. The supernatant (eluate) was then transferred to a new tube. The beads were washed with 200 μl of 10 mM Tris-HCl (pH 8.5), and the supernatant was also transferred to a new tube. Subsequently, 5 μl of proteinase K was added to the eluate and incubated for 6 h or overnight at 55 °C to reverse-cross-link the protein-DNA complexes. ChIP DNA was extracted with phenol:chloroform:isoamyl alcohol (Sigma-Aldrich, P3803), precipitated with ethanol, and resuspended in TE buffer. ChIP DNA libraries were prepared using an NEBNext® Ultra^™^ II DNA library prep kit for Illumina® sequencing (New England BioLabs, E7645). Briefly, ChIP DNA was end-repaired, ligated with an adaptor, and followed by 6–10 cycles of PCR amplification, per the manufacturer’s guidelines. Next, library fragments of 250–650 bp were selected using AMPure XP beads (Beckman, A63881). Finally, the DNA fragments were sequenced using an Illumina HiSeq X Ten system (paired-end 150-bp reads, Annoroad Gene Technology).

### Regular ChIP-Seq library preparation

The regular ChIP-Seq experiment was performed as reported^16^ with minor modifications. Young leaves were crosslinked using 1% formaldehyde and quenched with glycine. To isolate the nuclei or chromatin, approximately 1 g of young leaves were ground in liquid nitrogen into fine powder and resuspended in 30 ml of EB1 buffer. The mixture was filtered through Miracloth, and the filtrate was centrifuged at 1800 × g for 10 min at 4 °C. The pellet was washed three times in 1.5 ml of EB2 buffer and centrifuged at 2000 × *g* for 10 min at 4 °C. Next, the pellet was washed in 0.5 ml of EB3 buffer and centrifuged at 2000 × *g* for 1 h at 4 °C. The final pellet was resuspended in 0.25 ml of NLB buffer, and the chromatin was fragmented into 200–600 bp by sonication in a Bioruptor (Diagenode). The chromatin sample was then centrifuged at 2000 × *g* for 10 min at 4 °C, and the supernatant was used for IP. ChIP was performed using H3K4me3 antibody (Millipore, 07-473). The remaining steps, including washing the bead-IP complexes, ChIP DNA purification, library preparation, and DNA sequencing, were the same as described for eChIP-Seq.

### Comparison of efficiency between eChIP and regular ChIP

The protein-DNA complexes were reverse-crosslinked to purify DNA as described above, and the purified DNA was used to evaluate the chromatin content at distinct steps of the assays. The relative chromatin extraction efficiency at distinct steps was normalized to the amount of the starting material.

### ChIP-reChIP-Seq library preparation

Tissue fixing, chromatin sonication, and IP with the first antibody in the primary ChIP procedure were performed as described in eChIP. The first antibody was crosslinked to the beads using the fixative disuccinimidyl suberate (DSS, Pierce, 21555). After washing with low-salt ChIP buffer, high-salt ChIP buffer, ChIP wash buffer, and TE buffer, the protein-DNA complexes were eluted from beads by ChIP Elution buffer for 15 min at 65 °C with agitation at 900 rpm. The supernatant (eluted chromatin) was then transferred to a new tube. A total 5% of the eluted chromatin was retained for the primary ChIP DNA purification. The remaining eluted chromatin was then used for the reChIP. reChIP with the secondary antibody, elution, DNA purification, library construction, and sequencing were performed as described above.

### FAIRE-Seq library preparation

Open chromatin mapping was performed using the FAIRE-Seq (Formaldehyde-Assisted Isolation of Regulatory Elements) method^37,38^ with minor modifications for rice. Approximately 0.5 g of sample crosslinked with 1% formaldehyde was used for each experiment. Nuclei isolation was performed as described for regular ChIP-Seq. The isolated nuclei pellet was resuspended in 400 μl of Sonic-FAIRE buffer (10 mM Tris-HCl, pH 8.0, 100 mM NaCl, 1 mM EDTA, 0.5% SDS, and protease inhibitor cocktail) and fragmented into 200–400-bp pieces by sonication in a Bioruptor (Diagenode). Lysates were centrifuged at 20,000 × *g* for 10 min at 4 °C, and the supernatant was transferred to a new tube. To prepare FAIRE DNA, 1 volume of phenol:chloroform:isoamyl alcohol was added to the supernatant; the mixture was vortex-mixed and centrifuged at 20,000 × *g* for 10 min; and the supernatant was transferred to a new tube. These steps were repeated at least twice to remove nucleosome-coated DNA. After ethanol precipitation, FAIRE DNA was resuspended in TE buffer. Subsequently, DNA fragments of 200–400 bp were selected using AMPure XP beads. FAIRE DNA library preparation and sequencing were performed as described above for eChIP-Seq.

### RNA-Seq library preparation

Total RNA was isolated from the indicated tissues using a RNeasy plant mini kit according to the manufacturer’s instructions (QIAGEN, 74904). The RNA (2 μg) was used for library construction using an Illumina TruSeq RNA kit, according to the manufacturer’s recommendations. Sequencing was performed using an Illumina HiSeq X Ten system.

### Bisulfite-treated DNA sequencing

Genomic DNA was extracted using a DNeasy plant mini kit according to the manufacturer’s instructions (QIAGEN, 69104). Bisulfite conversion of DNA was conducted using the EZ DNA Methylation-GoldTM kit (ZYMO, D5005), and the bisulfite-treated DNA libraries were constructed using the Illumina TruSeq DNA sample prep kit, following the manufacturers’ instructions. Briefly, 2 μg of DNA for each sample was fragmented into 200–400-bp pieces by sonication in a Bioruptor (Diagenode). The fragmented DNA was processed by end-repair, 3′-end adenylation, and adapter ligation. The adapter-ligated DNA fragments were treated with bisulfite, followed by eight cycles of PCR amplification. Finally, the bisulfite-treated PCR products were purified using AMPure XP beads. The paired-end sequencing of bisulfite-treated DNA libraries was performed using an Illumina HiSeq X Ten system.

### Plasmid construction

To modify the reporter vector, the coding regions of the 35S promoter and attR1-ccdB-attR2 in the p2FLGW7 vector^39^ were replaced with a multiple clone site (MCS) and 35S terminator (35STor), respectively, to generate the pMCFLMC vector. The MCS fragment containing *Sac*I, *Nde*I, *Eco*RI, *Sph*I, *Hin*dIII, and *Spe*I restriction sites was prepared by annealing oligonucleotides. The 35STor fragment was amplified from p2FLGW7 with the 35STor forward and reverse primers, and *Kpn*I and *Bam*HI-*Xba*I-*Sal*I-*Pst*I-*Sac*II sites were added to the 5′ and 3′ ends, respectively. The attR1-ccdB-attR2 region of p2RLGW7^39^ was replaced with 35STor to generate p2RLMC. Then, DNA containing the assayed promoter region was amplified from MH63 genomic DNA using the corresponding primers; the amplified fragments were cloned into pMCFLMC for the promoter activity assay. The primers used in the current study are listed in Supplementary Table 4.

### Protoplast isolation and transient reporter assays

Protoplast isolation was performed as reported^40^ with minor modifications. Briefly, leaf sheaths of approximately 2-week-old MH63 seedlings were digested with 1.5% cellulase R-10 (Yakult Pharmaceutical, 150911-01) and 0.75% macerozyme R-10 (Yakult Pharmaceutical, 150108-01) for 4 h at room temperature. After being filtered through nylon mesh, the collected protoplasts were incubated in W5 solution (154 mM NaCl, 5 mM KCl, 125 mM CaCl_2_, and 22 mM MES, pH 5.7) for 1 h. The protoplasts were then collected and resuspended in MMG solution (0.6 M mannitol, 15 mM MgCl_2_, and 4 mM MES, pH 5.7). Plasmid pMCFLMC containing distinct promoter fragments and plasmid p2RLMC were used to cotransfect rice protoplasts using the PEG-CaCl_2_ method for 12–15 min. Next, the protoplasts were collected and incubated in WI solution (0.6 M mannitol, 4 mM KCl, and 4 mM MES, pH 5.7) in darkness for approximately 14 h. Finally, the protoplasts were lysed to perform a luciferase activity assay with the Dual Luciferase reporter assay system (Promega, E1910). The fLUC activity was normalized to the *Renilla* luciferase (rLUC) activity in each sample, and the relative ratio was determined by comparing with that of the empty vector. The mean relative ratios were calculated based on three independent experiments.

### Enhancer activity validation

The target DNA fragments were amplified from the genome DNA and then were recombined to linearized screening vector pBI221 with ClonExpress II One Step Cloning Kit at 37 °C for 30 min, followed by 4 °C for 10 min. All the constructs were introduced into Trans1-T1 Phage Resistant Chemically Competent Cells according to the manufacturer’s protocol. The transformants were transferred to 4 l LB medium (Amp^r^) for 13 h. The plasmid libraries were extracted using QIAGEN Plasmid Plus Kit. Protoplast isolation and protoplast transformation assay were performed as described above. Total RNA was isolated from the protoplasts using a RNeasy plant mini kit according to the manufacturer’s instructions (QIAGEN, 74904). First-strand cDNA synthesis was performed with TransScript One-Step gDNA Removal and cDNA synthesis SuperMix (AU311 50 °C for 30 min, 85 °C for 5 s) using a specific primer (GACTGGAGTTCAGACGTGTGC). All reactions were pooled. The first PCR (98 °C for 2 min, followed by 10−12 cycles of 98 °C for 30 s, 58 °C for 30 s, 72 °C for 30 s, and then 72 °C for 5 min), using two reporter-specific primers (R: 5′-GACTGGAGTTCAGACGTGTGC-3′ & F: 5′-GAGCTCTGGTAGAAATCTGAGGGTGTC-3′), one of which spans the splice junction of GFP intron. After cDNA synthesis using EasyScript One-Step gDNA Removal and cDNA Synthesis SuperMix (TransGen Biotech, AE311), the enhancer activities of the target DNA fragments were quantified using qRT-PCR. The primers used in the current study are listed in Supplementary Table 5.

### ChIP and FAIRE sequencing data analysis

Reads from eChIP-Seq libraries were aligned to the MH63/ZS97 assembly using bwa mem algorithm^41^. Mapped reads with a MAPQ quality score below 30 and PCR duplicates were filtered using SAMTools (1.3.1)^42^ to ensure high-quality aligned data. For analysis of H3K4me3, H3K27ac, and RNAPII libraries, narrow-peak calling settings were used in MACS2 (2.1.0)^43^: macs2 callpeak –t <input file>-c <control file>-f BAM –n <output peak file>-B -g 3.6e+8. For analysis of H3K9me2, H3K4me1, and H3K27me3 libraries, broad-peak mode was used in MACS2 with FDR < 0.1. RSC was set to >0.8, and NSC was set to >1.05 to assess the quality of each eChIP-Seq dataset; FRiP was used to assess the peak quality in the dataset^44^. For FAIRE-Seq data, two independent FAIRE-Seq libraries were created for each tissue or variety (MH63RS1 or ZS97RS1). The alignment process was similar to that for eChIP-Seq. FAIRE-Seq peaks were identified using MACS2 with the following settings: macs2 callpeak -t < input file>--nomodel--shift -100--extsize 200 -f BAM –n <output peak file>-B -q 0.05 -g 3.6e+8. Statistics for the data are provided in Supplementary Table 1. FAIRE-Seq and eChIP-Seq data were visualized using the IGV browser. Spearman’s correlation coefficient for normalized signals between two biological replicates of eChIP-Seq or FAIRE-Seq libraries was computed genome wide over 1-kb bins. Signal correlations were computed using all combinations of five histone modifications (H3K4me3, H3K27ac, H3K27me3, H3K4me1, and H3K9me2) and FAIRE-Seq data in four tissues and RNAPII in three tissues. The heatmap of correlations revealed high reproducibility between replicates. The high-quality replicates were merged, and respective peaks for subsequent analysis were identified. Fold-enrichment heatmaps and profiles of histone modifications were generated by deepTools software^45^. Signal levels were determined by FPKM (eChIP)/FPKM (input) for regions on both sides (2 kb) of genes, and data were sorted according to expression level (Supplementary Fig. 3). If there was a 1-bp overlap for the gene and our histone peaks, this gene was considered to be a corresponding gene of peaks. For ChIP-reChIP analysis, the steps of mapping were the same as the previous ChIP-Seq library, but using the first ChIP-Seq data as control.

### RNA-Seq analysis

Sequence quality of RNA-Seq libraries was evaluated using FastQC, and the adapter sequences and low-quality reads were filtered using Trimmomatic (0.36)^46^. The cleaned reads were mapped to the MH63 or ZS97 reference genome using TopHat2 (2.1.0)^46^, and gene expression was quantified using Cufflinks (2.2.1). Expressed genes were defined as those with FPKM > 1. Differentially expressed genes were evaluated using the DESeq2 package^47^ in R with an adjusted *p* value < 0.05 and log2 fold-change > 1.5.

### DNA methylation analysis

The sequence quality of the whole-genome bisulfite sequencing (WGBS) libraries was evaluated using FastQC, and the adapter sequences and low-quality reads were filtered using Trimmomatic. The cleaned reads were then mapped to the MH63/ZS97 reference genomes using BatMeth2^48^. The uniquely mapped reads were used for further analysis. Individual cytosines with coverage at least 3 were considered for methylation-level calling. The DNA methylation level of each cytosine was obtained by dividing the depth of methylated reads by the total coverage of individual cytosines.

### Analysis of differentially methylated regions

To identify the DMRs, the whole genome was divided into 200-bp bins. The DMR between two samples was examined by a binomial test and defined by two conditions: (1) the difference in DNA methylation between two samples was 0.6 or above, and (2) the adjusted *q* value between the average region methylation level was 0.05 or less.

### Characterization of chromatin states

ChromHMM v1.12^3^, a multivariate Hidden Markov Model, was used for unsupervised segmentation of the MH63 genome into a certain number of states based on the combination of histone modifications. The genome was divided into 200-bp bins. Since RNAPII is hard to detect in the root, five histone modifications, RNAPII, and FAIRE-Seq signals were selected in the three remaining tissues (young leaves, panicles, and mature leaves) to divide the MH63 genome into 15 states. Multiple models were trained on these data, with CS numbers ranging from 8 to 40. The 15-state model was chosen for all further analyses because it captured all the key information of CS. Greater numbers of CSs did not sufficiently capture significant information. In previous studies^12^, 15-state models were similarly trained on human tissue data.

### Correlation analysis between epigenome and transcription

Enrichment analysis of the overlap of different histone modification combinations with specific genomic features was performed using ChromHMM. Based on 200-bin segments in three tissues presented by ChromHMM, the expression level of each CS was evaluated by overlapping with genes. Similarly, the distribution of DNA methylation (percent CpG methylation from WGBS data) was computed using regions belonging to each of the 15 CSs based on the core set of six marks and FAIRE-Seq regions.

### Transposon elements analysis

MH63 and ZS97 TE annotations are available at the Rice Information GateWay^49^ (http://rice.hzau.edu.cn/rice/). ChIPseeker^50^ in the R package was used to analyze the genome-wide distribution of the three TE types (MITE, *Copia*, and *Gypsy*) and to combine different TE distributions with 15 core CSs, especially CS 13 (*Copia*-enriched state). CS 13 was mapped to a gene if it exhibited a 1-bp overlap with that gene and compared with the *Copia* distribution in CS 13. In the distribution comparison, if the intron and exon of the gene were simultaneously covered by TE, we defined TE as covering the exon region. The overall distribution of TEs close to histone modifications in the MH63 genome was visualized if 1-bp overlaps with peaks were observed, and different preferences were noted. To determine the methylation density of a TE region, methylation of all cytosine sites in the TE region was averaged. Genes with FPKM > 0.15 were analyzed to assess the relationship between different TE locations and the overlap of H3K9me2 with gene expression.

### Epigenome analysis around promoters

Considering the histone characteristics of the promoter region, unsupervised clustering of four histone modifications and open chromatin signals around TSS (2 K) in young leaves was performed. The analysis revealed 15 typical categories of histone combinations around TSS. Histone signal level was determined based on the FPKM (eChIP)/FPKM (input) ratio every 10 bp in the vicinity of TSS. Each cluster contained at least 2000 genes and showed a unique gene expression and classification. These clusters were also relatively stable in other tissues. Further, DNA methylation profiles on both sides of TSS were evaluated using these clustering genes. The results corresponded to the histone pattern (Supplementary Fig. 7).

The data revealed different modification patterns in the vicinity of TSS in rice, including the blank cluster 10 corresponding to the quiescent state. Genes in clusters 1 and 2 (with many active histone modifications) had very high expression levels and genes in cluster 13 (modified by H3K9me2) were located in the heterochromatic region. It is worth noting that cluster 4 marked the boundary between euchromatin and heterochromatin, with the transition from high-H3K9me2 and low-H3K4me3 profile to low-H3K9me2 and high-H3K4me3 profile.

### Chromatin state variability and switch frequency

Using the 15-state model, the entire genome was divided into 200-bp segments, and the switching counts for any pair of states in all pairs of epigenomes in three different tissues were computed. For example, for a given pair of states A and B, the numbers of genomic bins that were marked as (A, B) or (B, A) in all pairs of epigenomes were counted. A 15 × 15 relative state-switching matrix was computed for every two-tissue combination. The switching matrix was transposed and summed with the original matrix to obtain a symmetric matrix. To obtain the overall conversion frequency in the three tissues, the switching matrices of each pair of tissues were added. The switching matrix (which was symmetric) was then row-normalized to convert the switching counts to switching probabilities. This was done to avoid dependence on the numbers of different CSs.

### DNA methylation variation in chromatin state switching

As a general resource of epigenomics, the comparison of all epigenomes is available for methylation data. The DMR resolution was set to 200 bp. The same process as in CS variability and switching was used to construct the transformation matrix to combine the changes in the overall genome DMR regions with histone modifications. Each element in the DMR matrix was divided by the number of CS transitions, relative DMR switching counts was obtained, and the matrix was row-normalized. The above calculation method (CG switching matrix) was used to process the CHG and CHH DMR switching matrices. The three kinds of DMRs exhibited the same pattern throughout the histone change at the overall level. There was a very small chance of a DMR relative occurring if a histone was not modified. However, some CSs switched in the respective DMRs of CHH, CG, and CHG had particular features.

### Differential analysis of each histone mark

Histone modification regions were defined based on the merged peak for each duplicate between tissues. The differential histone signal was also calculated using the DESeq2 package in R; an adjusted *p* value of 0.05 and log2 fold-change of >1.5 were used to find significantly differential histones. A comparison of peaks between any two tissues is shown in Supplementary Table 2.

### Characterizing histone marks and transcription in tissues

Since changes in some histone modifications in tissues are not obvious, only three histones (H3K4me3, H3K27ac, and H3K27me3) were selected for further dynamic analysis. Using the distribution of different histones, if a 1-bp overlap existed between a histone and a promoter region of a gene, the changes in histone and gene pairs between any two tissues were identified. Among these, H3K4me3 showed a positive correlation (*R* > 0.52) with the expression level in tissues, in addition to young leaves and mature leaves, and the same was observed for H3K27ac (*R* > 0.3). However, H3K27me3 showed a negative correlation with gene expression in different tissues.

RNA-Seq data for 20 tissues (Supplementary Table 3) were analyzed to evaluate the expression breadth of histone-marked genes. Histone-marked genes, which were not exclusive, were defined by BEDTools (2.25.0)^51^ if they overlapped the histone peaks, and all RNA-Seq data were aligned to the MH63 genome. The expressed genes were defined as those with FPKM > 1.

### Tissue-specific histone modification analysis

To identify tissue-specific peaks in the different tissues, regions of different peaks in any two tissues were extracted and merged at first. Then, the signals of the differential peak regions in the four tissues were row-normalized. At last, the subsequent unsupervised clustering revealed four tissue-specific clusters. The corresponding genes in each cluster were identified using the above method of GO enrichment; only the first ten significant features were selected (Fig. 4d).

### Candidate enhancer analysis

FAIRE-Seq peaks were divided into two types DRE and PRE. All low-methylation and no DNA methylation regions that overlapped with open chromatin regions, excluding the ones overlapping with genes and promoter regions, were predicted to be DRE. Since DREs were located within the intergenic regions, a joint analysis of histone modifications (H3K27ac, H3K9me2, H3K4me3, H3K4me1 and H3K27me3) and candidate regions was performed by deepTools (2.5.3). DREs were linked to putative target genes based on nearby genes. Then, FAIRE-Seq signals of DRE were clustered in the three tissues. Based on these clusters, expression levels of the putative target genes were examined and found to be consistent with the determined clustering patterns. In order to analyze the PRE, we used the H3K4me3 loop from the H3K4me3 ChIA-PET data^27^, and genes were divided into six groups by the way whether there is a loop or overlapping with proximal FAIRE-Seq peaks.

### Dynamic of epigenome feature analysis in 20 rice varieties

All histone mark libraries were aligned to the Nipponbare (MSU v7.0) genome. Histone modification signal differences (fold-change > 1.5, *p* value < 0.05) between each variety pair were compared using DESeq2. The percentages of histone differences among 20 varieties were compared. We randomly extract ten comparing combinations to simulate the saturation curve from each histone mark. Genes were divided into two categories, core (candidate core) genes and distributed genes, based on the annotations of core (candidate core) genes and distributed genes downloaded from the pan genome website (http://cgm.sjtu.edu.cn/3kricedb).

### Influence of genetic variations on the epigenomes

The distribution of SNPs within 2 kb on each side of a histone modification region in 10-bp units was calculated. To identify the boundaries between SNPs more precisely and to exclude the peak length interferences, the 1-kb region before the peak region was divided into 20 units, and the density of SNPs was calculated. We used the same CS model as MH63 (Fig. 2a) to train data from all the varieties to form the same CS as before, and we calculate the conserved regions which the CS had no changes in 20 rice varieties (each CS number was normalized by proportion in genome). We ensure that the CS of the nine *Xian*/*Indica* varieties different from the CS of the two *Geng*/*Japonica* varieties to filter candidate different regions between *Xian*/*Indica* and *Geng*/*Japonica*. Through these different regions, we have calculated the similarity between other subgroups (Aus, ARO and Int) with *Xian*/*Indica* or *Geng*/*Japonica* which is also in the 200-bp resolution. The CS resolution was set to 200 bp when we calculated the CS transfer from *Xian*/*Indica* to *Geng*/*Japonica* and heatmap of switch matrix was normalized by proportion of CS in genome. We computed each CS genome coverage of 20 varieties and normalized by *Z* score to get the genome coverage plot. NMDS plot was used, vegan R package and epigenetic signals were counted in 20 rice varieties.

### Reporting summary

Further information on research design is available in the Nature Research Reporting Summary linked to this article.

## Supplementary information

Supplementary Information

Reporting Summary

## Data Availability

Data supporting the findings of this work are available within the paper and its Supplementary Information files. A reporting summary for this article is available as a Supplementary Information file. The datasets generated and analyzed during the current study are available from the corresponding authors upon request. The sequence data are available at NCBI GEO under accession number GSE142570. All accessions of published RNA-Seq data used in this study are provided in Supplementary Table 3. The source data underlying Figs. 1f, g, 2k−n, 3b, d, 4c−f, h, 5c−e, g, 6a, h–j, l, as well as Supplementary Figs. 3, 4, 6d, f, 11, 12f, 14a, 15a, c, d, and 16 are provided as a Source Data file.

## Source data

Source Data
