# Supplementary material for: Integrative analysis of reference epigenomes in 20 rice varieties
Source: Nat Commun. 2020 May 27;11:2658. doi: 10.1038/s41467-020-16457-5 (PMC7253419; doi:10.1038/s41467-020-16457-5)
Supplement: Supplementary file 1 — Supplementary Information [file 41467_2020_16457_MOESM1_ESM.pdf]

Integrative analysis of reference epigenomes in 20 rice varieties

Zhao *et al.*

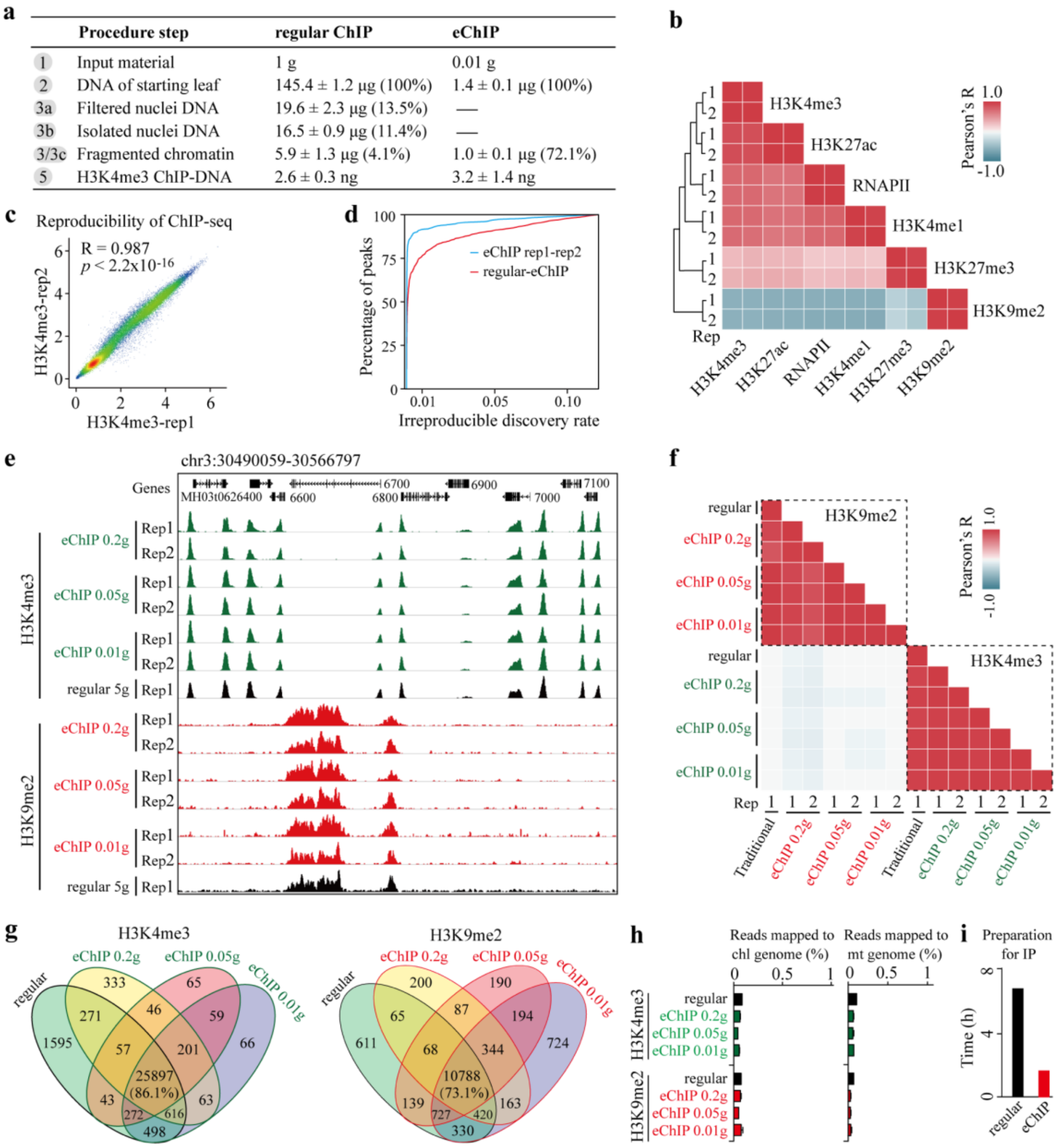

Supplementary Fig. 1. eChIP as a fast and robust method for low-input samples in rice.

a, Comparison of chromatin extraction efficiency in different steps of regular ChIP and eChIP in Fig. 1a. The protein-DNA complexes were reverse–cross-linked, and the purified DNA was used to evaluate chromatin content in different steps. Relative chromatin extraction efficiency at the different steps was normalized to the amount of the starting material. Each value represents the mean ± standard error of mean (n = 3 biological replicates). **b**, Genome-wide correlation heat map (with bin size 1,000 bp) for eChIP-Seq data of two biological replicates across different histone marks and different sample amounts. **c**, Pearson correlation for two replicates of H3K4me3 eChIP-Seq. Numbers of the mapped reads from each genomic bin (10 kb) were plotted in log scale between two replicates. The R value calculated by Pearson correlation coefficient at 10 kb

genomic bin is shown. p-values were calculated by comparing a fitted model to a null model using the ‘anova’ function; $p < 2.2 \times 10^{-16}$.**d**, Irreproducible discovery rate analysis for eChIP-Seq and regular ChIP-Seq of H3K4me3. **e**, Genome browser screenshot showing regular ChIP (5 g input material) and eChIP (0.01–0.2 g input material) datasets from young leaves of rice. **f**, Genome-wide Pearson correlation heat map (with 1-kb bin size) between H3K4me3 and H3K9me2 with regular ChIP and eChIP data for different sample amounts. **g**, Reproducibility of peaks called by regular ChIP and eChIP. Venn diagrams display a high percentage of overlap between peaks called by the indicated methods generated from different sample amounts. **h**, Comparison of the percentages of reads mapped to chloroplast (Chl) or mitochondrion (mt) genome from the regular ChIP and eChIP. **i**, Comparison of IP preparation time for regular ChIP and eChIP.

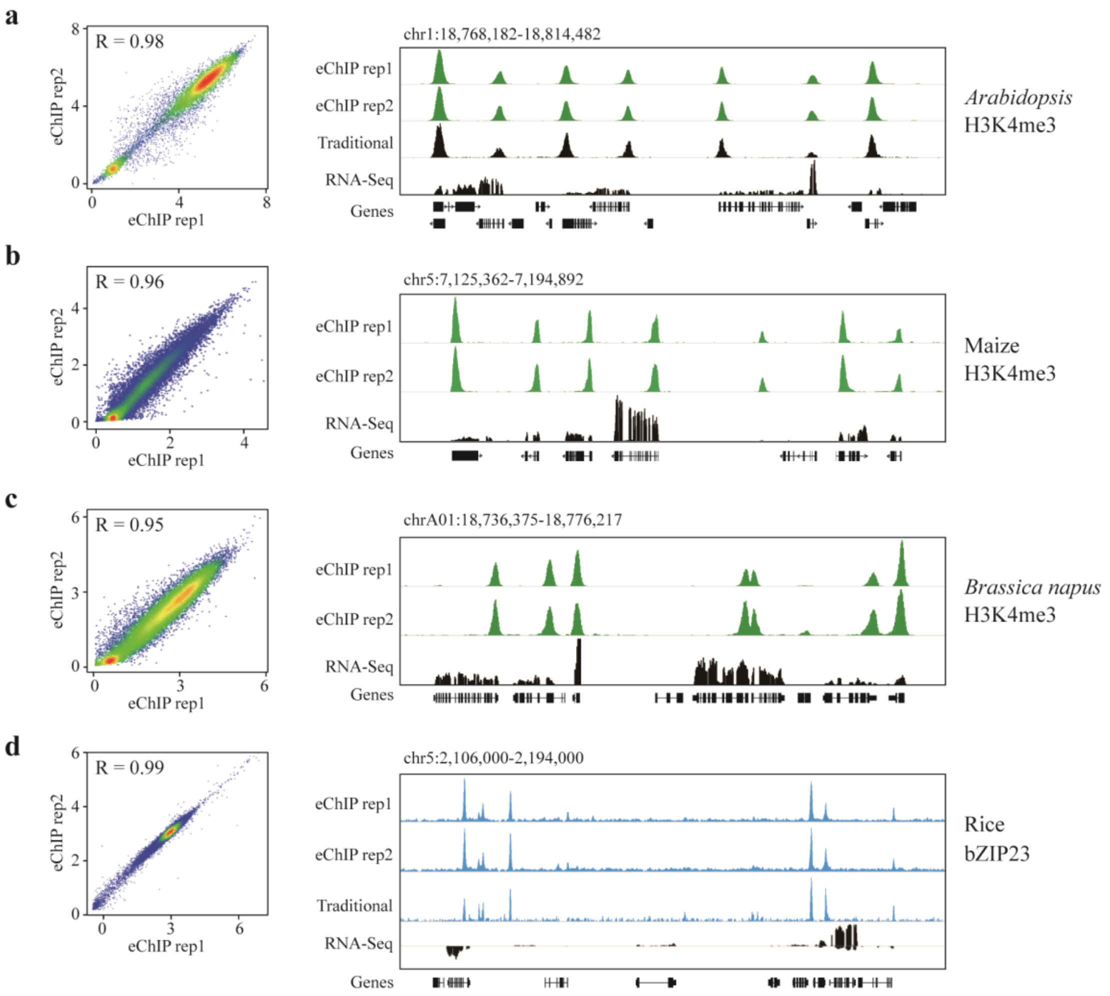

Supplementary Fig. 2. eChIP works for modified histones and transcription factor in the indicated plant species.

a–**d**, Pearson correlation for two replicates of H3K4me3 eChIP-Seq data from young leaves of *Arabidopsis* (**a**), Maize (**b**), *Brassica napus* (**c**), and transcription factor (bZIP23) eChIP-Seq data from young leaves of rice (**d**). Representative genome browser screenshots for ChIP-Seq data were also shown.

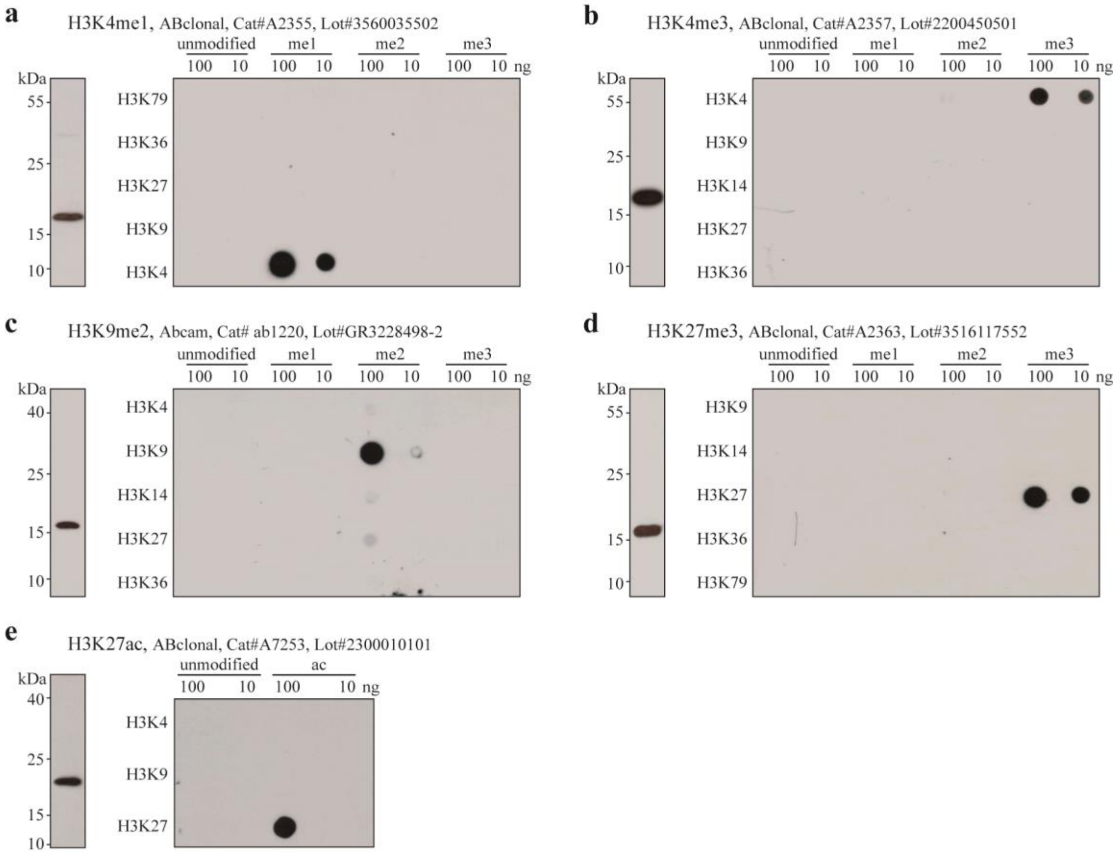

Supplementary Fig. 3. Antibody specificity validation.

a–e, Western blots of the indicated histone modification antibodies recognizes a ~17 kDa protein in rice protein lysate (left panel). Dot blots confirmed specificity of modified histones (right panel). The experiments were repeated two times with similar results. Source data are provided as a Source Data file.

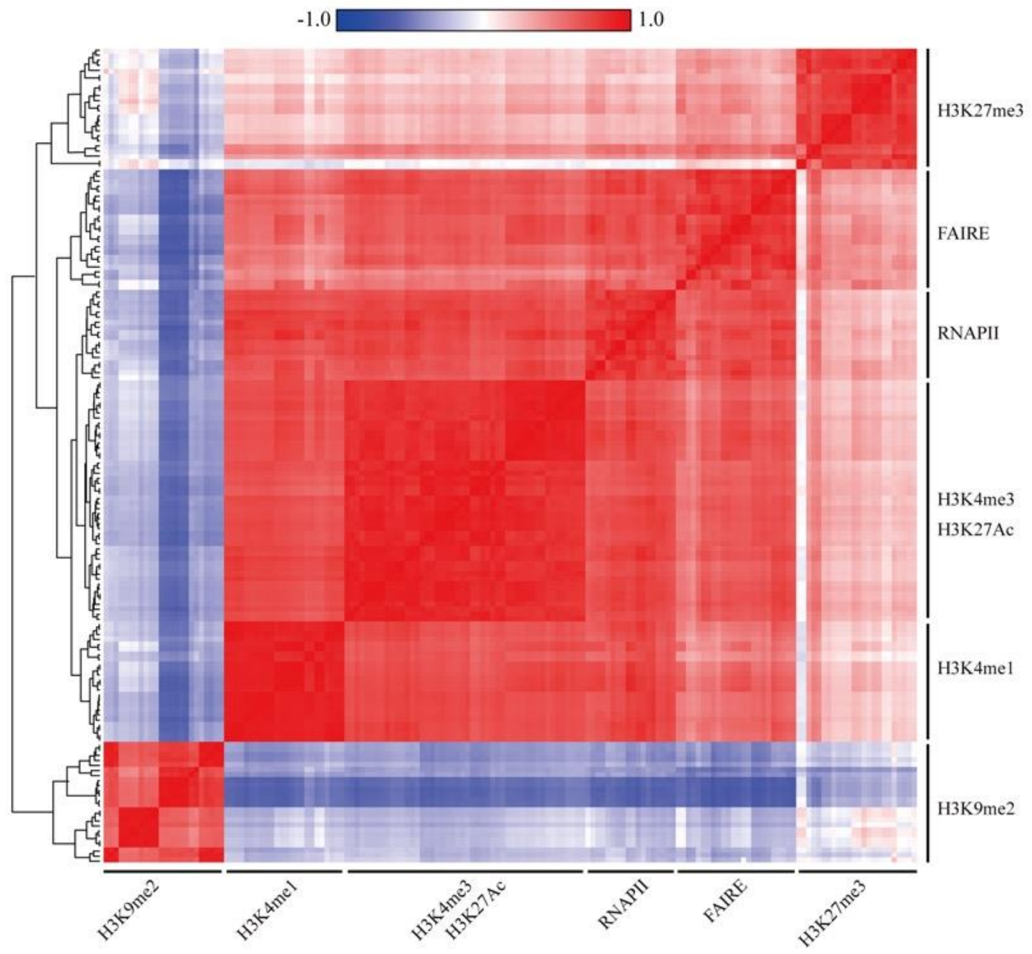

Supplementary Fig. 4. Correlation analysis for epigenomic datasets in rice.

Genome-wide correlation heatmap (with 10-kb bin size) for eChIP-Seq and FAIRE-Seq datasets across different rice tissues and varieties (MH63, ZS97, and Nip) used in this study. Source Data are provided as a Source Data file.

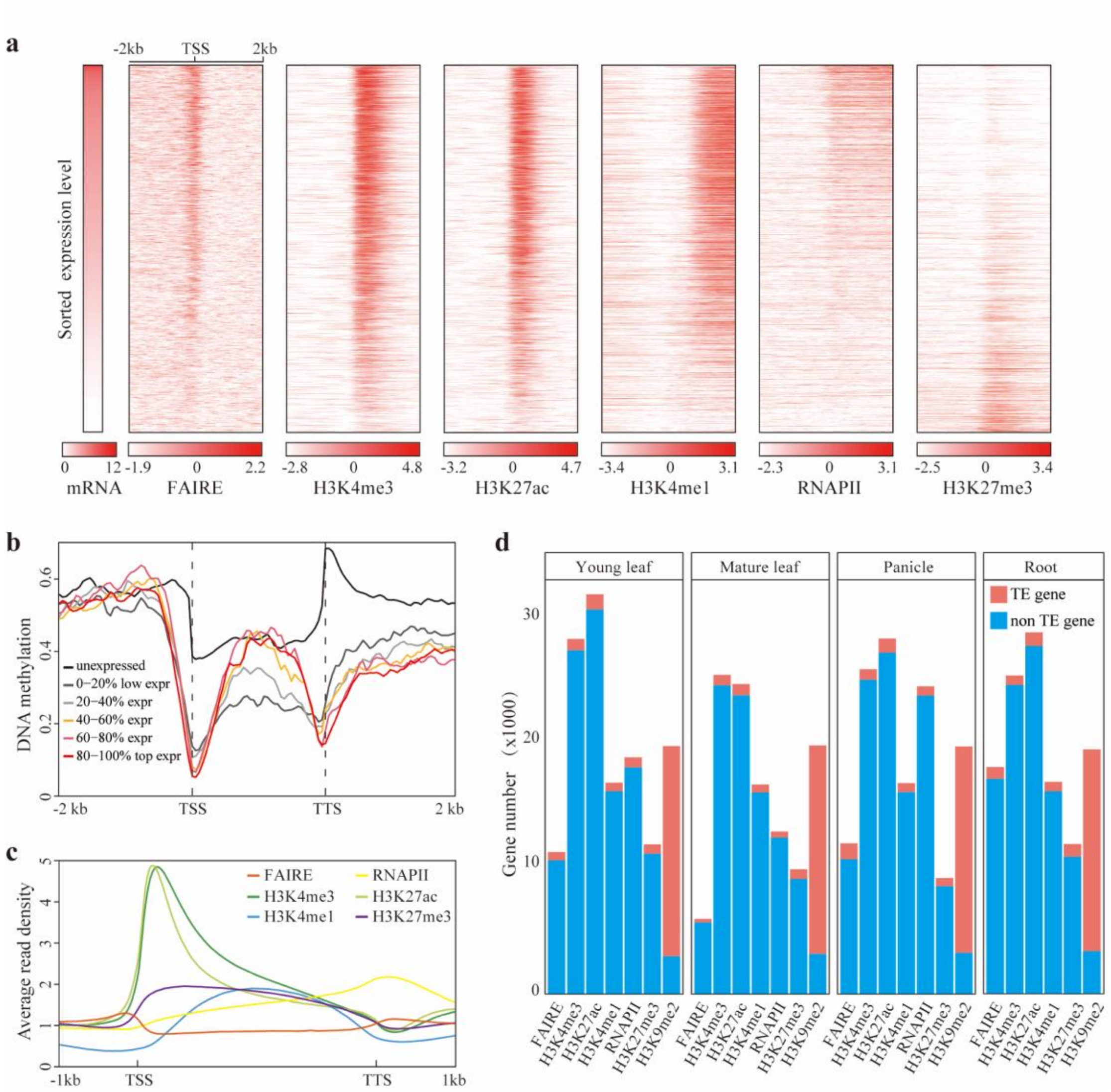

Supplementary Fig. 5. The association among epigenomic modifications, transcription activity, TE genes and non-TE genes in MH63.

a, Heatmap of epigenetic marks and RNAPII occupancy on all annotated rice genes, which were sorted according to their expression level determined by RNA-Seq. For each gene, the histone mark intensity is displayed along ± 2 kb regions around TSS. **b**, DNA methylation levels for genes showing different expression levels. The y-axis indicates the sample sizes used in the analysis. **c**, Distribution of histone marks, open chromatin (FAIRE), and RNAPII occupancy along genes in young leaves. The gene body was converted into percentiles to standardize genes with different lengths. Regions 1 kb upstream and downstream of the gene are shown. **d**, Number of TE and non-TE genes associated with histone marks, open chromatin, and RNAPII occupancy in the indicated tissues.

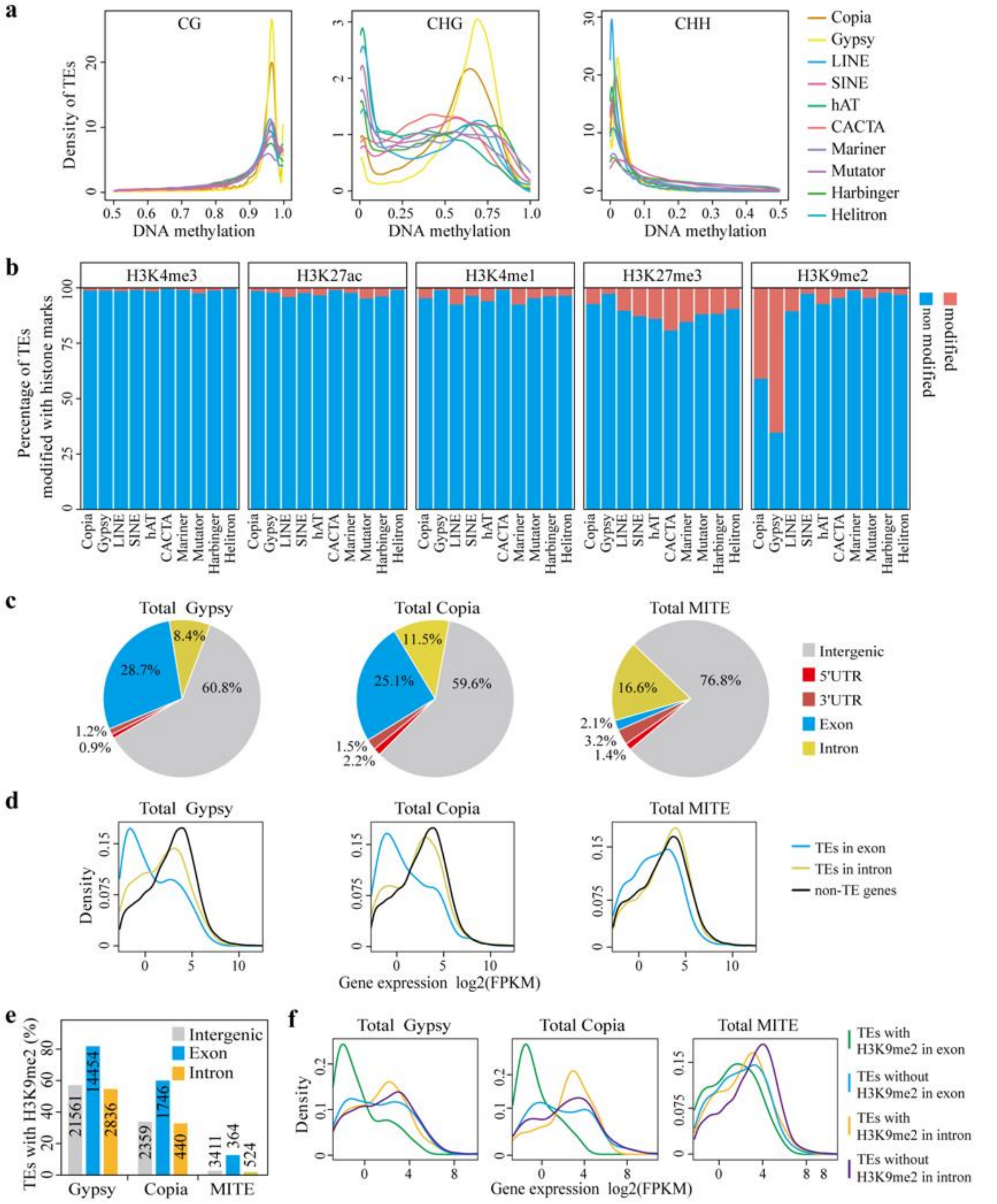

Supplementary Fig. 6. DNA methylation and histone modifications of TEs and the effect on gene transcription in young leaves of MH63.

a, Distribution of methylation levels for retrotransposons and DNA transposons. **b**, Percentage of TEs modified by different histone marks. A TE overlapped 1 bp with the histone peak was treated as this histone mark-modified TE. The y-axis indicates the sample sizes used in the analysis. **c**, Distribution of TEs in different regions of the rice genome. MITE includes hAT, CACTA, Mariner, Mutator, Mim, and Harbinger DNA transposons. **d**, Probability density curve of gene expression. TE genes associated with different TEs located in the exon or intron, and non-TE genes are shown.

Genes associated with TEs in exons have higher proportion with low expression. Retrotransposons (*Gypsy* and *Copia*) were more likely to insert into exons than were DNA transposon MITEs. Genes with TEs located in exons had a larger proportion of unexpressed genes than did genes with TE located in introns and with no MITE. **e**, H3K9me2 modification rate of TEs located in different regions of the rice genome. **f**, Probability density curve of gene expression. TE genes motioned in (**e**), with or without H3K9me2, are shown. TE genes associated with H3K9me2-modified exons have higher proportion with low expression. Source Data underlying Supplementary Figure 6d and 6f are provided as a Source Data file.

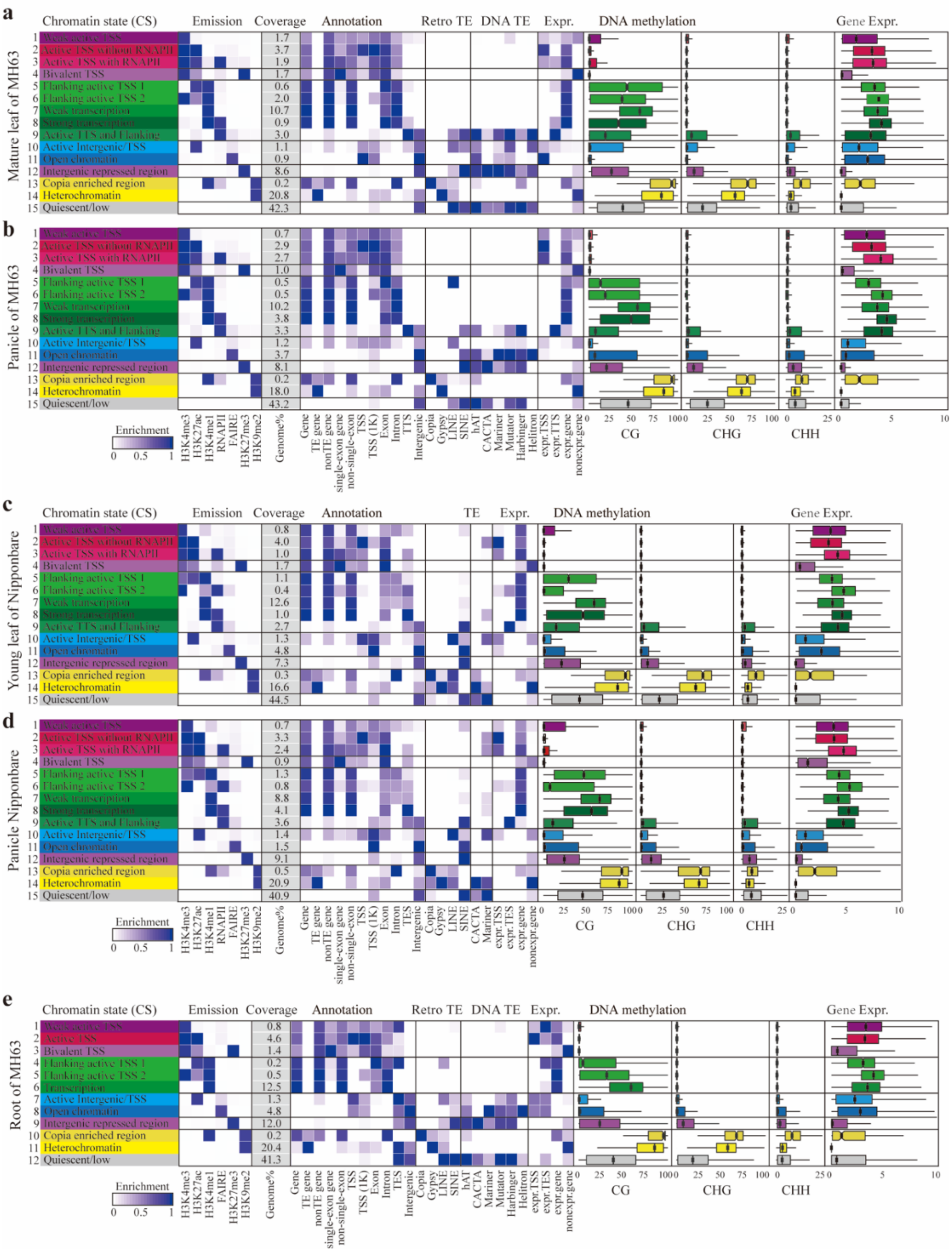

Supplementary Fig. 7. Chromatin state (CS), TE enrichment, DNA methylation, and gene expression for different rice tissues from MH63 and Nipponbare.

a–d, Enrichments for 15-state model based on five histone marks, FAIRE, and RNAPII occupancy in the indicated tissues and varieties. **e,** Enrichments for 12-state model based on five histone marks and FAIRE in root. Cov, genome coverage; Expr, enrichments of expression. Boxplots in **a-e** include a median with quartiles and outliers above the top whisker. Coverage in **a** showed the total bin size in whole genome, which indicate the sample sizes used in the analysis.

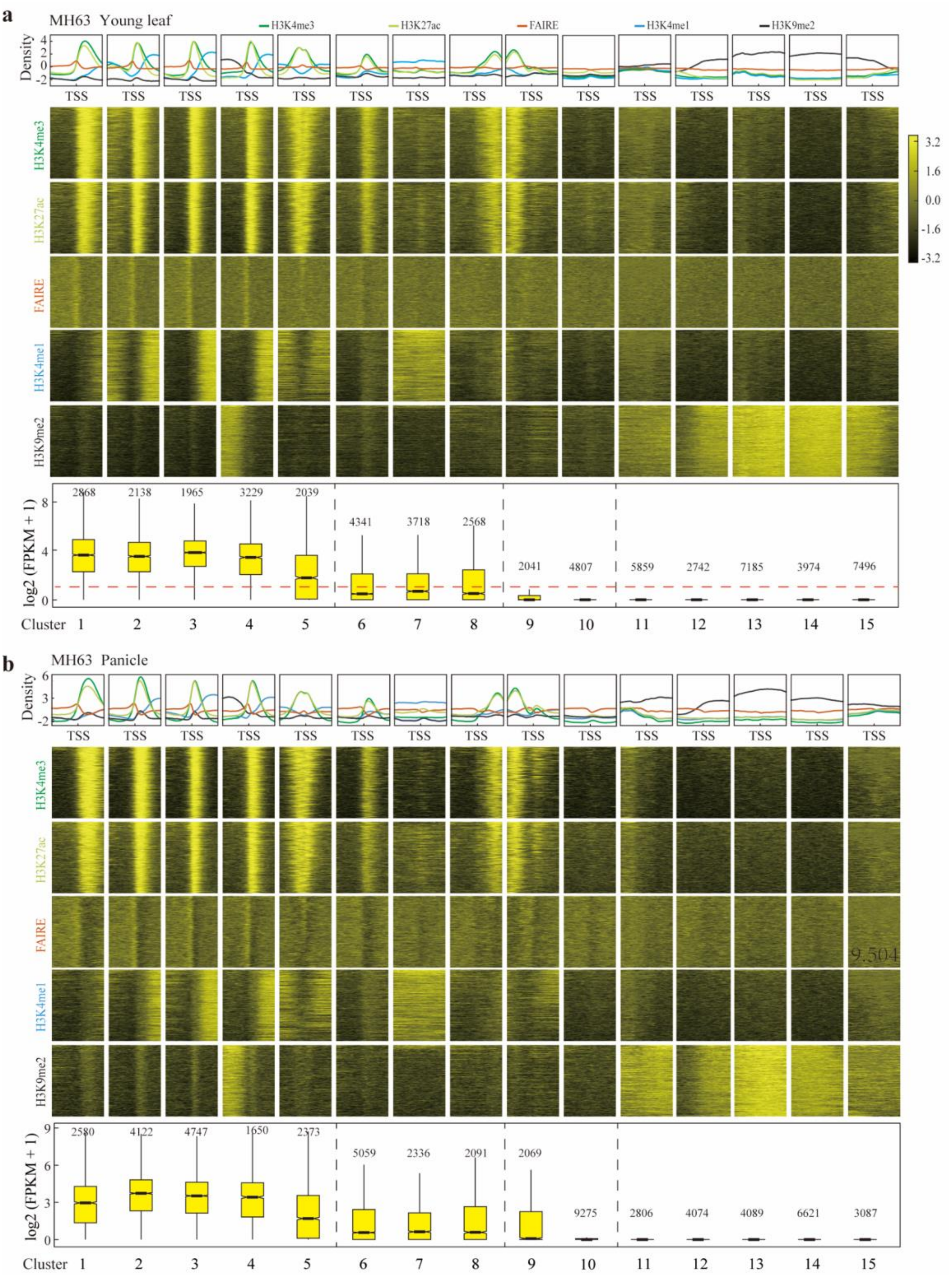

Supplementary Fig. 8. Epigenetic features and transcription activities of promoters in MH63.

a, **b**, 15 clusters of promoters defined by four histone marks and open chromatin in the indicated tissue, and transcriptional activity of genes in each cluster. Boxplots in **a**, **b** include a median with quartiles and outliers above the top whisker. The numbers indicate sample size used in the analysis.

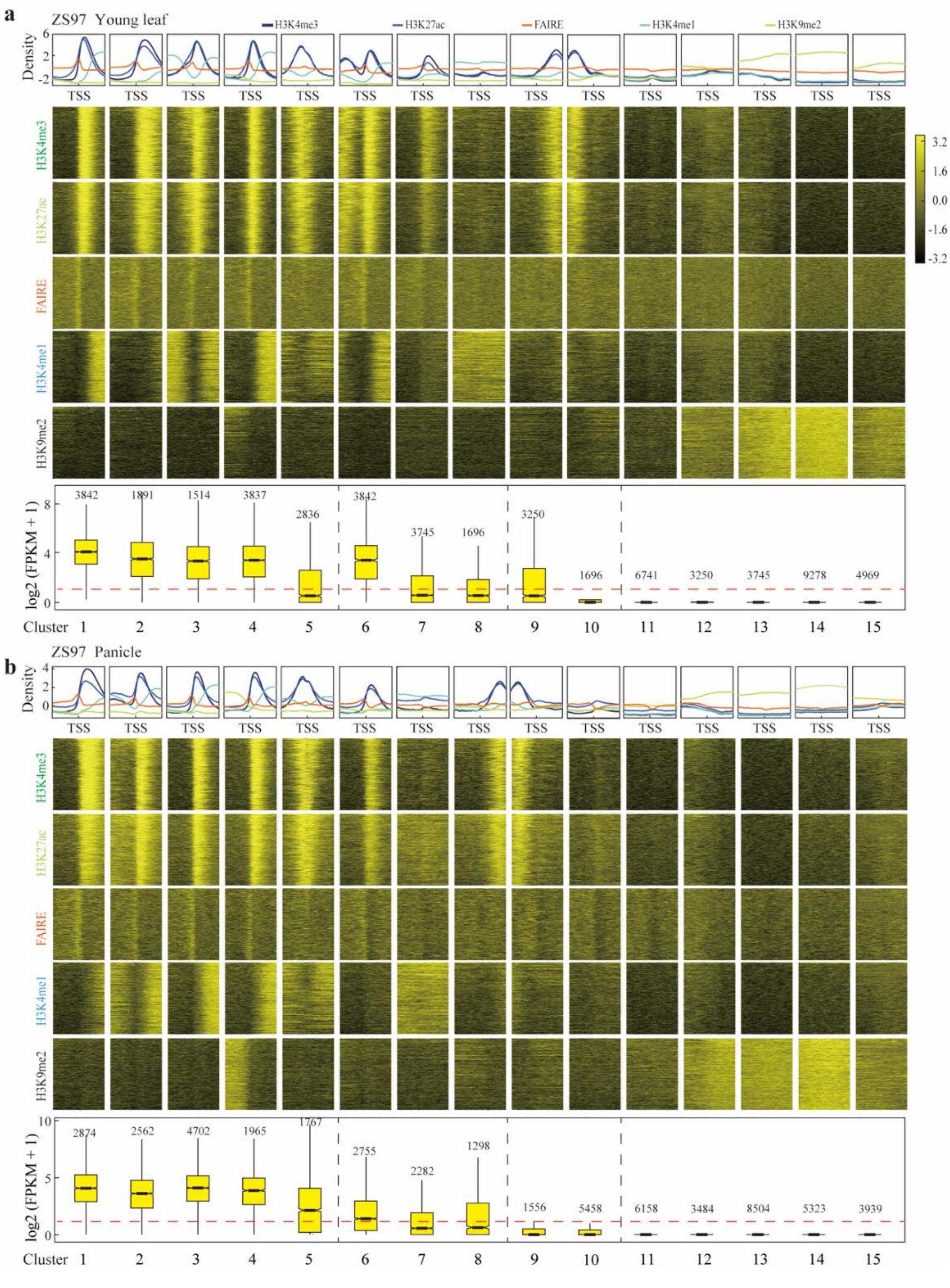

Supplementary Fig. 9. Epigenetic features and transcription activities of promoters in ZS97. **a**, **b**, 15 clusters of promoters defined by four histone marks and open chromatin in the indicated tissue, and transcriptional activity of genes in each cluster. Boxplots in **a**, **b** include a median with quartiles and outliers above the top whisker. The numbers indicate sample size used in the analysis.

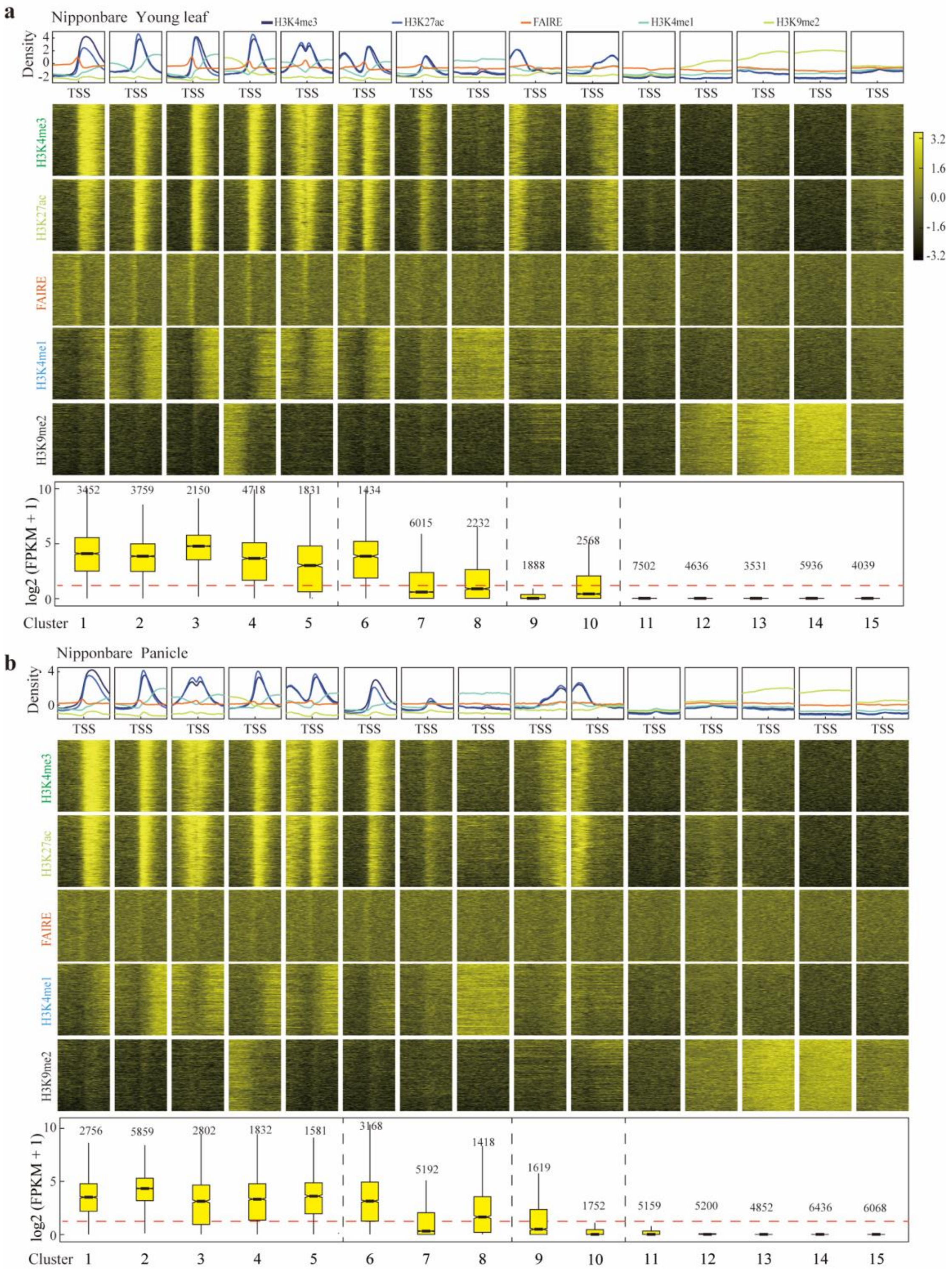

Supplementary Fig. 10. Epigenetic features and transcription activities of promoters in Nipponbare.

a, **b**, 15 clusters of promoters defined by four histone marks and open chromatin in the indicated tissue, and transcriptional activity of genes in each cluster. Boxplots in **a**, **b** include a median with quartiles and outliers above the top whisker. The numbers indicate sample size used in the analysis.

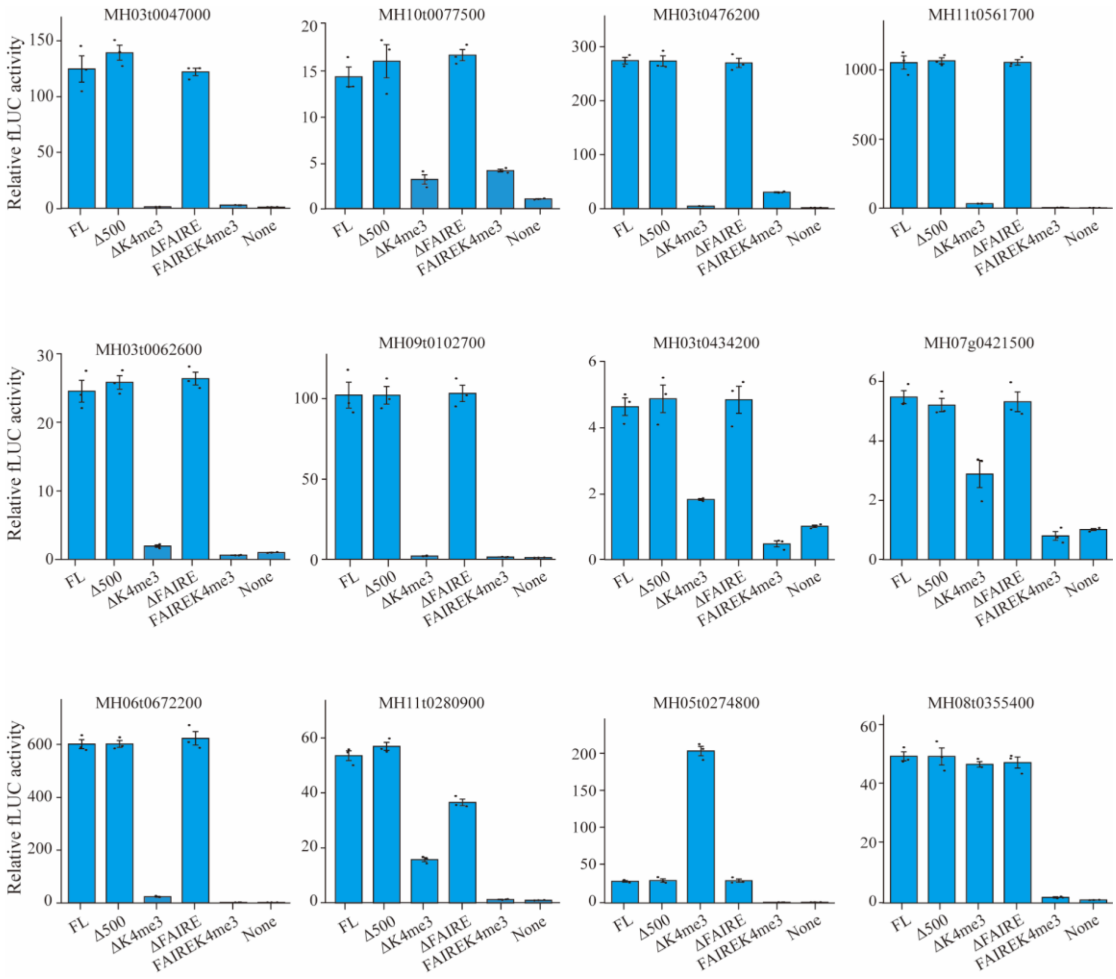

Supplementary Fig. 11. The effect of FAIRE and H3K4me3-marked promoter regions on transcription.

Similar to the descriptions in Fig. 3b, a transient reporter assay was used to examine the effects of different promoter regions on gene expression in rice protoplasts. The relative fLUC activities from 12 randomly selected genes are shown. Empty vector served as the negative control. The data indicate the relative luminescence level. Each value represents the mean ± standard error of mean (n = 3 biological replicates). Source data are provided as a Source Data file.

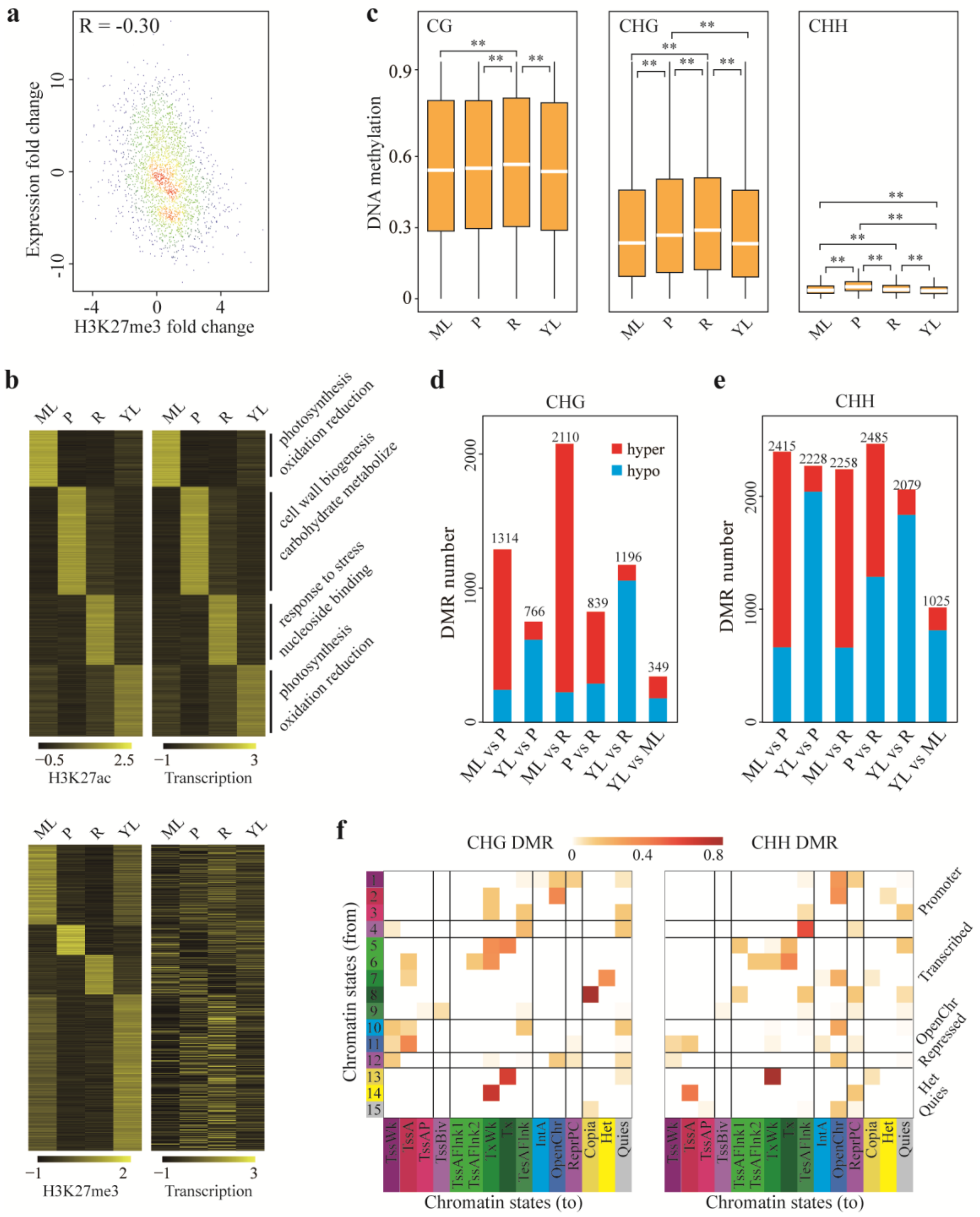

Supplementary Fig. 12. Dynamics of epigenetic marks and gene expression in MH63 tissues.

a, Correlations of log2 fold-change in H3K27me3 and gene expression between mature leaf and panicle. **b**, Dynamics of histone marks and transcription in four tissues. Heatmaps of tissue-specific histone marks and gene expression levels are presented. Biological GO functions enriched for differentially regulated genes with elevated expression are shown. ML, mature leaf; P, panicle; R, root; YL, young leaf. **c,** Comparison of DNA methylation levels in different tissues. The whole genome was divided into 200-bp bins to calculate the DNA methylation levels.

Boxplots in **c** show the median, third and first quartiles. ** $p < 0.01$ from Wilcoxon test. The numbers indicate the sample size used in the analysis. **d**, **e**, Numbers of CHG and CHH DMRs in different tissues. **f**, Log10 ratio of relative CG and CHH DMR switch frequency with which a region switches from one chromatin state (row) to another (column). Source Data underlying Supplementary Figure 12f is provided as a Source Data file.

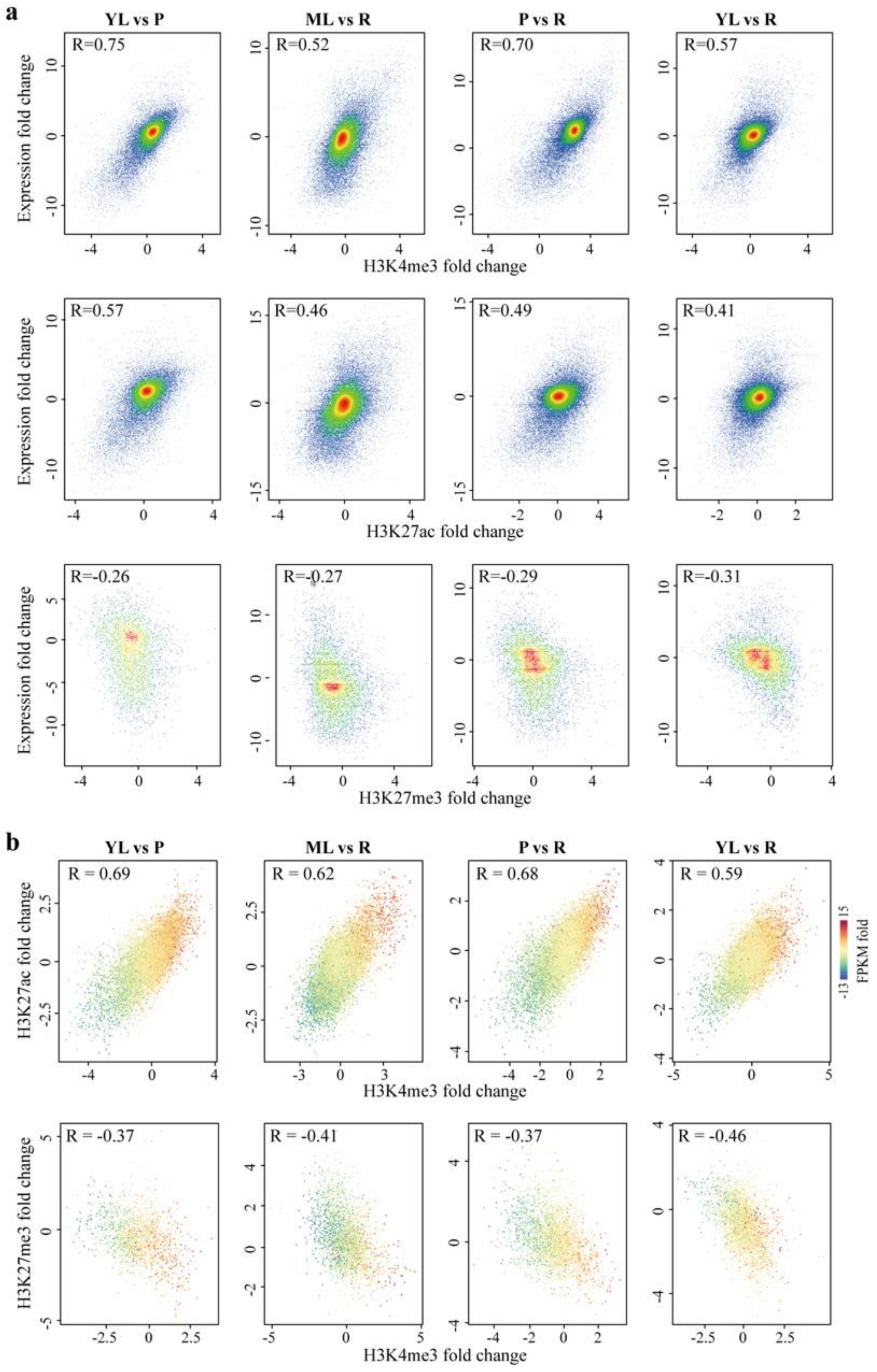

Supplementary Fig. 13. Dynamics of histone marks and gene expression in different tissues. **a**, Correlations of log2 fold-changes in a single histone mark and gene expression between the indicated tissues. **b**, Correlations of log2 fold-changes in H3K4me3 and H3K27ac (or H3K27me3)

between the indicated tissues. Color scale indicates the fold-changes in gene expression. Only data of genes with both H3K4me3 and H3K27ac (or H3K27me3) marks are plotted here. ML, mature leaf; P, panicle; R, root; YL, young leaf.

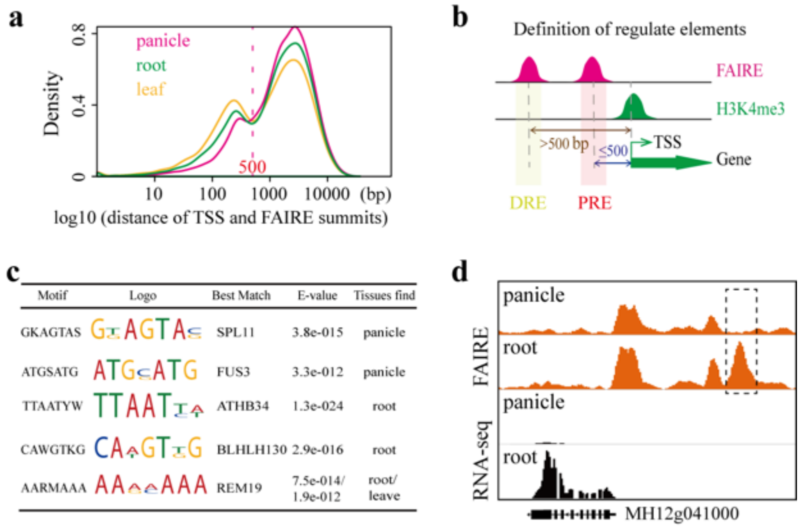

Supplementary Fig. 14. FAIRE-defined distal and proximal DNA regulatory elements.

a, Density distribution of the distance between FAIRE summits and TSS. Red dotted line represents the position of 500 bp upstream of TSS. **b**, Schematic diagram for definition of distal regulatory element (DREs) and proximal regulatory element (PREs). **c**, Motifs identified in the tissue-specific DREs. **d**, An example showing that the expression levels of *MH12g041000* proximal to the DRE (dotted box) was enhanced in the root, but not in the panicle. Source Data underlying Supplementary Figure 14a is provided as a Source Data file.

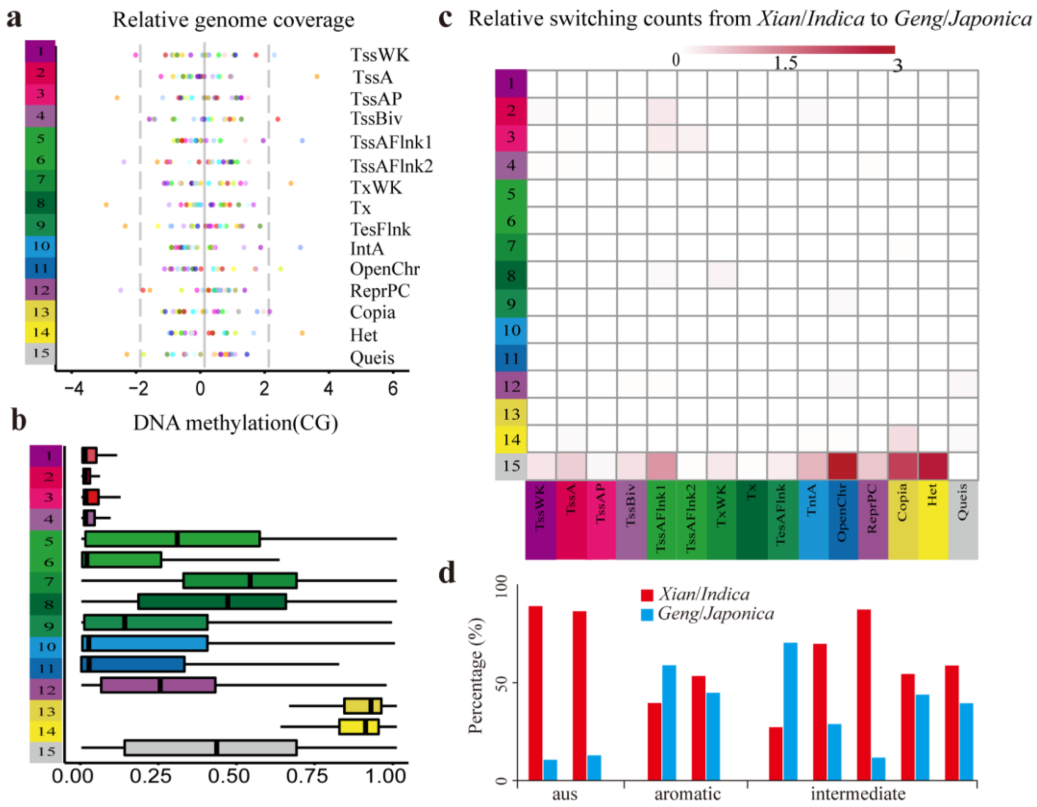

Supplementary Fig. 15. Rice varieties differences in chromatin states.

a, Relative chromatin state frequency for each reference epigenome in 20 rice varieties. **b**, DNA methylation level of chromatin state, labeled with each state. **c**, Relative switch frequency of chromatin states from *Xian*/*Indica* to *Geng*/*Japonica.* Boxplots in **b** show the median , third and first quartiles. **d.** Trends of chromatin state in Aus, ARO (aromatic) and Int (intermediate) subpopulations. Only *Xian*/*Indica* and *Geng*/*Japonica* different regions about 45000 bins (per 200 bp) are considered. Source Data underlying Supplementary Figure 15a, 15c, and 15d are provided as a Source Data file.

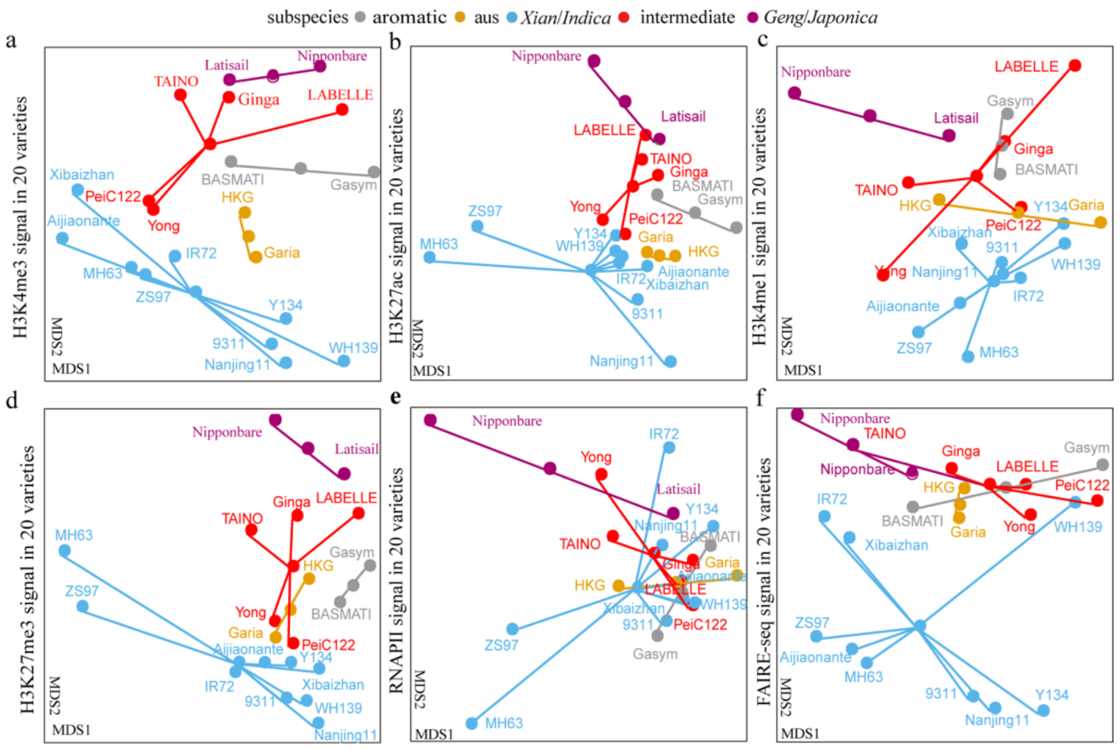

Supplementary Fig. 16. NMDS analysis for Epigenetic signal in 20 rice Varieties.

a–**f**, Non-metric Multidimensional scaling (NMDS) plots indicate the relationships of 20 rice varieties based on similarity in each epigenetic signal. First two dimensions are shown as MDS1 versus MDS2. Source Data are provided as a Source Data file.

Supplementary Table 1. Summary of regular ChIP-seq and eChIP-seq data from rice young leaves.

Antibody	Sample title	Raw data	Clean data	Mapped reads	Mapping rate	Peak No.	FRIP	NSC	RSC
**H3K4me3**	eChIP 0.2g Rep 1	32,380,414	30,234,556	29,227,622	96.67%	27,626	78.63%	1.60	1.03
	eChIP 0.2g Rep 2	25,292,790	24,096,580	22,547,309	93.33%	25,327	77.94%	1.71	1.05
	eChIP 0.05g Rep 1	38,662,656	36,898,890	34,500,250	93.50%	26,324	85.80%	1.79	1.13
	eChIP 0.05g Rep 2	63,664,220	57,977,455	55,277,397	95.34%	25,267	90.55%	2.19	1.07
	eChIP 0.01g Rep 1	29,618,844	28,252,006	26,254,860	92.93%	27,491	88.36%	1.85	1.11
	eChIP 0.01g Rep 2	27,242,970	23,976,505	22,508,421	93.88%	25,384	87.83%	1.99	1.10
	regular ChIP	27,828,322	26,562,340	25,078,347	94.41%	29,901	83.93%	1.70	1.01
**H3K9me2**	eChIP 0.2g Rep 1	67,743,714	61,472,015	60,988,077	99.21%	11,341	72.65%	1.04	1.00
	eChIP 0.2g Rep 2	26,368,192	25,353,242	25,103,775	99.02%	12,079	80.72%	1.06	1.06
	eChIP 0.05g Rep 1	21,092,424	19,494,763	19,209,170	98.54%	11,016	73.10%	1.05	1.22
	eChIP 0.05g Rep 2	44,774,648	40,367,759	38,729,736	95.94%	13,457	84.86%	1.08	1.05
	eChIP 0.01g Rep 1	36,291,684	34,669,137	31,375,536	90.50%	12,719	73.86%	1.05	0.90
	eChIP 0.01g Rep 2	44,774,648	27,490,715	26,143,784	95.10%	13,895	80.04%	1.06	0.99
	regular ChIP	50,162,278	47,874,510	44,671,828	93.31%	14,779	61.38%	1.05	0.79

FRiP, fraction of reads in peaks; NSC, normalized strand coefficient; RSC, relative strand correlation.

Supplementary Table 2. Summary of rice epigenome datasets generated in this study.

Tissue	Variety	eChIP-Seq	FAIRE -Seq	RNA -Seq	WGBS	low input eChIP	regular ChIP-Seq	ChIP-re ChIP	
Young leaf	MH63	12	2	2	1	12	2	6	
Young leaf	ZS97	12	2	2	1				
Young leaf	Nip	12	2	2	1				
Young leaf	Aijiaonante	12	2	2	1				
Young leaf	PeiC122	12	2	2	1				
Young leaf	Xibaizhan	12	2	2	1				
Young leaf	WH139	12	2	2	1				
Young leaf	9311	12	2	2	1				
Young leaf	Nanjing11	12	2	2	1				
Young leaf	Gasym Hany	12	2	2	1				
Young leaf	HKG 98	12	2	2	1				
Young leaf	Yong Chal Byo	12	2	2	1				
Young leaf	TAINO 38	12	2	2	1				
Young leaf	Y134	12	2	2	1				
Young leaf	IR72	12	2	2	1				
Young leaf	Latisai1	12	2	2	1				
Young leaf	Ginga	12	2	2	1				
Young leaf	Garia	12	2	2	1				
Young leaf	LABELLE	12	2	2	1				
Young leaf	BASMATI 385	12	2	2	1				
Panicle	MH63，ZS97，Nip	36	6	6	4				
Root	MH63，ZS97，Nip	30	6	6	4				
Mature leaf	MH63，ZS97，Nip	36	6	6	4				
Total		342	58	58	32	12	2	6	510

eChIP-Seq (including two regular ChIP-Seq) data of five histone modification marks (H3K4me3, H3K27ac, H3K4me1, H3K27me3, and H3K9me2), RNA polymerase II (RNAPII), and transcription factor. WGBS, whole-genome bisulfite sequencing. Each experiment has two biological replications.

Supplementary Table 3. RNA-Seq data for different tissues used in this study.

Tissue number	Tissue	SRR accession	data link	Varieties
1	Young leaf (two-week -old)	SRR10751 892	https://www.ncbi.nlm.nih.gov/sra?term=SRX7426703	*Xian*/*Indica* (MH63, this study)
		SRR10751 893	https://www.ncbi.nlm.nih.gov/sra?term=SRX7426704	
2	Flag leaf before flower transition	SRR10751 898	https://www.ncbi.nlm.nih.gov/sra?term=SRX7426709	
		SRR10751 899	https://www.ncbi.nlm.nih.gov/sra?term=SRX7426710	
3	Panicle (1.5-4.5 cm)	SRR10751 894	https://www.ncbi.nlm.nih.gov/sra?term=SRX7426705	
		SRR10751 895	https://www.ncbi.nlm.nih.gov/sra?term=SRX7426706	
4	One-week-old root	SRR10751 896	https://www.ncbi.nlm.nih.gov/sra?term=SRX7426707	
		SRR10751 987	https://www.ncbi.nlm.nih.gov/sra?term=SRX7426708	
5	Four-week -old root	SRR51340 63	https://www.ncbi.nlm.nih.gov/sra/?term=SRR5134063	*Geng*/*Japonica* (Chilbo)
		SRR51340 64	https://www.ncbi.nlm.nih.gov/sra/?term=SRR5134064	
6	Calli (embryonic stage)	SRR37246 15	https://www.ncbi.nlm.nih.gov/sra/?term=SRR3724615	*Geng*/*Japonica* (TNG67)
		SRR37246 16	https://www.ncbi.nlm.nih.gov/sra/?term= SRR3724616	*Xian*/*Indica* (IR64)
7	Endosperm	SRR31234 79	https://www.ncbi.nlm.nih.gov/sra/?term= SRR3123479	Hybrid of *Xian*/*Indica* (Longtefu) and *Geng*/*Japonica* (02428)
		SRR31234 81	https://www.ncbi.nlm.nih.gov/sra/?term= SRR3123481	
8	Nodes I and II	SRR17772 39	https://www.ncbi.nlm.nih.gov/sra/?term= SRR1777239	*O. sativa*
		SRR17772 40	https://www.ncbi.nlm.nih.gov/sra/?term= SRR1777240	*O. sativa*

Supplementary Table 3. RNA-Seq data for different tissues used in this study (continued).

Tissue number	Tissue	SRR accession	data link	Varieties
9	Stem	SRR1777241	https://www.ncbi.nlm.nih.gov/sra/?term= SRR1777241	*O. sativa*
		SRR1777242	https://www.ncbi.nlm.nih.gov/sra/?term= SRR1777241SRR1777242	*O. sativa*
10	Flower buds before flowering	SRR1213690	https://www.ncbi.nlm.nih.gov/sra/?term= SRR1213690	*Geng*/*Japonica* (Nipponbare)
11	Flowers at the flowering day	SRR1213691	https://www.ncbi.nlm.nih.gov/sra/?term= SRR1213691	
12	Milk grains	SRR1213696	https://www.ncbi.nlm.nih.gov/sra/?term SRR1213696=	
13	Mature seeds	SRR1213697	https://www.ncbi.nlm.nih.gov/sra/?term= SRR1213697	
14	Panicle (0.3-1.5 cm)	SRR1633182	https://www.ncbi.nlm.nih.gov/sra/?term= SRR1633182	*Geng*/*Japonica* (DongJin)
		SRR1633187	https://www.ncbi.nlm.nih.gov/sra/?term= SRR1633187	
15	Egg cell	SRR976335	https://www.ncbi.nlm.nih.gov/sra/?term= SRR976335	*Geng*/*Japonica* (Kitaake)
		SRR976336	https://www.ncbi.nlm.nih.gov/sra/?term= SRR976336	
		SRR976337	https://www.ncbi.nlm.nih.gov/sra/?term= SRR976337	
16	Sperm cell	SRR976338	https://www.ncbi.nlm.nih.gov/sra/?term= SRR976338	
		SRR976339	https://www.ncbi.nlm.nih.gov/sra/?term= SRR976339	
		SRR976340	https://www.ncbi.nlm.nih.gov/sra/?term= SRR976340	
17	Vegetative cell	SRR976341	https://www.ncbi.nlm.nih.gov/sra/?term= SRR976341	
		SRR976342	https://www.ncbi.nlm.nih.gov/sra/?term= SRR976342	
		SRR976343	https://www.ncbi.nlm.nih.gov/sra/?term= SRR976343	

Supplementary Table 3. RNA-Seq data for different tissues used in this study (continued).

Tissue number	Tissue	SRR accession	data link	Varieties
18	Three-week-old leaf	SRR711322	https://www.ncbi.nlm.nih.gov/sra/?term= SRR711322	*Geng*/*Japonica* Nipponbare
		SRR711323	https://www.ncbi.nlm.nih.gov/sra/?term= SRR711323	
19	Three-week-old calli	SRR358795	https://www.ncbi.nlm.nih.gov/sra/?term= SRR358795	
		SRR358797	https://www.ncbi.nlm.nih.gov/sra/?term= SRR358797	
20	Lamina joints of flag leaf	SRR976168	https://www.ncbi.nlm.nih.gov/sra/?term= SRR976168	

Supplementary Table 4. Constructs and primer sequences used in this study.

Target	Construct	Forward primer	Reverse primer
pMCFLMC/p2RLMC	35S terminator	GGGGTACCCGGCCATGCTAGAGTCCG	TCCCCGCGGCTGCAGGTCGACTCTAGAGGATCCAGGTCACTGGATTTTGGTTT
	MCS	GGAATTCCATATGGAATTCGCATGCAAGCTTACTAGTCC	GGACTAGTAAGCTTGCATGCGAATTCCATATGGAATTCC
MH07t0100900 promoter	FL	GGAATTCCATATGCTATTGGGTATGTGGCATCCATAAC	CCCAAGCTTCACAAACAAACAAACCACACAACC
	Δ500	GGAATTCCATATGATACTTGCCGAGTTTTTATACGAATGC	CCCAAGCTTCACAAACAAACAAACCACACAACC
	ΔK4me3	GGAATTCCATATGATACTTGCCGAGTTTTTATACGAATGC	CCCAAGCTTGGTTGGGTTTCGTTCACCTCG
	FAIREK4me3	GGAATTCCATATGGAAAAACAACAATGAAATAAACAGAGAA	CCCAAGCTTCACAAACAAACAAACCACACAACC
	ΔFAIRE	GGAATTCCATATGCCGGCGAGGTGAACGAAACC	CCCAAGCTTCACAAACAAACAAACCACACAACC
MH03t0047000 promoter	FL	GGAATTCCATATGCCTCCCTCTAAACTTGATACCCCG	GGAATTCGCCCGTCAGGATTTCATCGTAC
	Δ500	GGAATTCCATATGGTTATTTGTTGGGGATTGGGATCTA	GGAATTCGCCCGTCAGGATTTCATCGTAC
	ΔK4me3	GGAATTCCATATGGTTATTTGTTGGGGATTGGGATCTA	GGAATTCATGGCTGGCTGGTCTGCGAAG
	FAIREK4me3	GGAATTCCATATGAATAGTGTAATTGTACATCAAACGTATAA	GGAATTCGCCCGTCAGGATTTCATCGTAC
	ΔFAIRE	GGAATTCCATATGTTCGCAGACCAGCCAGCCAT	GGAATTCGCCCGTCAGGATTTCATCGTAC

Supplementary Table 4. Constructs and primer sequences used in this study (continued).

MH10t0077500 promoter	FL	GGAATTCCATATGTTCAGGACCTAGATATCACACCCGC	CCCAAGCTTACAACCCCGCAAATCGCCTAAAC
	Δ500	GGAATTCCATATGCAGATGACAAGGGAACATTCCTTTTG	CCCAAGCTTACAACCCCGCAAATCGCCTAAAC
	ΔK4me3	GGAATTCCATATGCAGATGACAAGGGAACATTCCTTTTG	CCCAAGCTTGGGAGGAGGTGGAGAGCTTCTAGA
	FAIREK4me3	GGAATTCCATATGCGGTGAAATATACCGCAGAGTATTT	CCCAAGCTTACAACCCCGCAAATCGCCTAAAC
	ΔFAIRE	GGAATTCCATATGTCCTCCCATGGCGGCGACGA	CCCAAGCTTACAACCCCGCAAATCGCCTAAAC
MH03t0476200 promoter	FL	GGAATTCGGGGTCGCTGGAGATGGAGACG	CCCAAGCTTATGCGACCAAGGCGAGGGGA
	Δ500	GGAATTCTCGTTGATGTTACAGTGTCGCATGATG	CCCAAGCTTATGCGACCAAGGCGAGGGGA
	ΔK4me3	GGAATTCTCGTTGATGTTACAGTGTCGCATGATG	CCCAAGCTTGCATGTGGGCCTCCCGTCGC
	FAIREK4me3	GGAATTCTTACCAAAACGCTCTTACAAGGTCT	CCCAAGCTTATGCGACCAAGGCGAGGGGA
	ΔFAIRE	GGAATTCCGAGGGAAGCGAAGGTAAGGAA	CCCAAGCTTATGCGACCAAGGCGAGGGGA
MH11t0561700 promoter	FL	GGAATTCTTTTGTCGCCTATTTTTTCACGG	CCCAAGCTTACATACCTTGCCATCGACTTCCC
	Δ500	GGAATTCGATATATGCACGTGTTCTCCTGGTGA	CCCAAGCTTACATACCTTGCCATCGACTTCCC
	ΔK4me3	GGAATTCGATATATGCACGTGTTCTCCTGGTGA	CCCAAGCTTGGTGCCGCCTCTTCAAATCTCTCC
	FAIREK4me3	GGAATTCGGTTTTTCTTTTCTTTTTCCGCACT	CCCAAGCTTACATACCTTGCCATCGACTTCCC
	ΔFAIRE	GGAATTCCTGGGCGTTTTCCGTGTTTGAAT	CCCAAGCTTACATACCTTGCCATCGACTTCCC

Supplementary Table 4. Constructs and primer sequences used in this study (continued).

MH03t0062600 promoter	FL	GGAATTCCTTCAGTTCACCCAAAAAAAATCCT	CCCAAGCTTAATTAAATCCGGACCACACAAGTTT
	Δ500	GGAATTCCCTCTCTGGCACAACTGACCTTG	CCCAAGCTTAATTAAATCCGGACCACACAAGTTT
	ΔK4me3	GGAATTCCCTCTCTGGCACAACTGACCTTG	CCCAAGCTTAGCTGACGTGAACCATCGTCGC
	FAIREK4me3	GGAATTCTGAAGAGGAGGAGCCGCAAGAA	CCCAAGCTTAATTAAATCCGGACCACACAAGTTT
	ΔFAIRE	GGAATTCGCCTTACCTGTTGTGTTCTTCGTC	CCCAAGCTTAATTAAATCCGGACCACACAAGTTT
MH09t0102700 promoter	FL	GGAATTCCATATGTAACGCCACCAACCTCCTCACTATT	CCCAAGCTTAACCACAACCAAGAAACCGCCC
	Δ500	GGAATTCCATATGTTTCTATTTTCAGGATAAACCATGTCGG	CCCAAGCTTAACCACAACCAAGAAACCGCCC
	ΔK4me3	GGAATTCCATATGTTTCTATTTTCAGGATAAACCATGTCGG	CCCAAGCTTTCGCGATTCCTCTCTCCGTCTC
	FAIREK4me3	GGAATTCCATATGTCTCCGTGAAAAAACAATATGATG	CCCAAGCTTAACCACAACCAAGAAACCGCCC
	ΔFAIRE	GGAATTCCATATGCCGACCCTCCTCTCGTCCTATC	CCCAAGCTTAACCACAACCAAGAAACCGCCC
MH03t0434200 promoter	FL	CGAGCTCGTAGATCCTTGTACCAGCGTACCC	GGAATTCTAAAAAGTTCCTACCAGAATCGCC
	Δ500	CGAGCTCGAAGGAGGGAGTACTTGTCAA	GGAATTCTAAAAAGTTCCTACCAGAATCGCC
	ΔK4me3	CGAGCTCGAAGGAGGGAGTACTTGTCAA	GGAATTCGCTTGAGGGAAACCATCGCC
	FAIREK4me3	CGAGCTCTTTTGTGTGAATGACTAATGGGTCC	GGAATTCTAAAAAGTTCCTACCAGAATCGCC
	ΔFAIRE	CGAGCTCCTACCGTATGATGTTCTGTGCCTGC	GGAATTCTAAAAAGTTCCTACCAGAATCGCC

Supplementary Table 4. Constructs and primer sequences used in this study (continued).

MH07g0421500 promoter	FL	GGAATTCTTGGTTAAAATTATTTCTAGGGCTCTG	CCCAAGCTTTCAGGATTGGACAACATAAAACACC
	ΔΔ500	GGAATTCTTTTTGGTTTGACTGATATGTGGAT	CCCAAGCTTTCAGGATTGGACAACATAAAACACC
	ΔK4me3	GGAATTCTTTTTGGTTTGACTGATATGTGGAT	CCCAAGCTTCATGGCTGGATTAGCTCGGTAG
	FAIREK4me3	GGAATTCTTTTTGGTTTGACTGATATGTGGAT	CCCAAGCTTTCAGGATTGGACAACATAAAACACC
	ΔFAIRE	GGAATTCATTCCTGAAGGTCTGCGTGCTA	CCCAAGCTTTCAGGATTGGACAACATAAAACACC
MH06t0672200 promoter	FL	GGAATTCCATATGCTGCACATATATACACAGCGGGC	CCCAAGCTTGCTAACCCCTAACCCGAAACGAT
	Δ500	GGAATTCCATATGATATTTTCATTAAATCTATAATTGAATAAT	CCCAAGCTTGCTAACCCCTAACCCGAAACGAT
	ΔK4me3	GGAATTCCATATGATATTTTCATTAAATCTATAATTGAATAAT	CCCAAGCTTGGCTGACCGGATCGGCGAGGGC
	FAIREK4me3	GGAATTCCATATGTACCATCCATCTCGCTTTCTGC	CCCAAGCTTGCTAACCCCTAACCCGAAACGAT
	ΔFAIRE	GGAATTCCATATGACGAGGTATGCTGCTGTGCTGTGTG	CCCAAGCTTGCTAACCCCTAACCCGAAACGAT
MH11t0280900 promoter	FL	GGAATTCCATATGCCCAGTTTTTTTCCCCTGCATGT	GGAATTCCTACCAGATCCGATCATACTGAGCG
	Δ500	GGAATTCCATATGGAAGCAGCTCCTTCTTGCGGAT	GGAATTCCTACCAGATCCGATCATACTGAGCG
	ΔK4me3	GGAATTCCATATGGAAGCAGCTCCTTCTTGCGGAT	GGAATTCAAACACAACGATCTGGCGCGGA
	FAIREK4me3	GGAATTCCATATGGTTGAGGCATACGATTTAGATTAGC	GGAATTCCTACCAGATCCGATCATACTGAGCG
	ΔFAIRE	GGAATTCCATATGCCGGATCCACTCAACCCCCC	GGAATTCCTACCAGATCCGATCATACTGAGCG

Supplementary Table 4. Constructs and primer sequences used in this study (continued).

MH05t0274800 promoter	FL	GGAATTCCATATGCCGCCCTCTCGCTAGATCCAGA	CCCAAGCTTATAAGCAAATCTATGACACCACTACAG
	Δ500	GGAATTCCATATGACTGAGAACGCTCTCGGTTTAGAT	CCCAAGCTTATAAGCAAATCTATGACACCACTACAG
	ΔK4me3	GGAATTCCATATGACTGAGAACGCTCTCGGTTTAGAT	CCCAAGCTTCTTGTTGGATTGCAGCTAGGGCC
	FAIREK4me3	GGAATTCCATATGACTGAGAACGCTCTCGGTTTAGAT	CCCAAGCTTATAAGCAAATCTATGACACCACTACAG
	ΔFAIRE	GGAATTCCATATGCCCCAGGTCCTTTTCTCATCAC	CCCAAGCTTATAAGCAAATCTATGACACCACTACAG
MH08t0355400 promoter	FL	GGAATTCCATATGTGGCCTAAAGCAGATTCTAGTATTAAAC	CCCAAGCTTAAAGAGTCCATCAGTAATAAGCCCA
	Δ500	GGAATTCCATATGTTAATCTCAAAACATGATGCTCAGG	CCCAAGCTTAAAGAGTCCATCAGTAATAAGCCCA
	ΔK4me3	GGAATTCCATATGTTAATCTCAAAACATGATGCTCAGG	CCCAAGCTTCGAGACGAGACAAGAGGAGGAAC
	FAIREK4me3	GGAATTCCATATGAATATTGTTAAGGTTTATTTTGGTTACA	CCCAAGCTTAAAGAGTCCATCAGTAATAAGCCCA
	ΔFAIRE	GGAATTCCATATGGAAGAAGTGTGCGTACTACTGCGT	CCCAAGCTTAAAGAGTCCATCAGTAATAAGCCCA

Supplementary Table 5. Primer sequences used for enhancer validation in this study.

	Target	Construct		qRT-PCR	
		**Forward primer**	**Reverse primer**	**Forward primer**	**Reverse primer**
Enhancer-like promoter	1: MH11g0492200	ACACTCTTTCCCTACACGACGGCACACGACACGAGCACGAA	GACTGGAGTTCAGACGTGTGCTCTCTCTACCTCTTCTTGAGCCTAA	GTACAACACTCTTTCCCTACACGAC	GAGGAAGTGGACTGCGAGTT
	2: MH02g0167000	ACACTCTTTCCCTACACGACGCCAATCTAAGAACAAGAAGAGATCC	GACTGGAGTTCAGACGTGTGCTTTTTCGCACTGTCATATTGAATAC	GTACAACACTCTTTCCCTACACGAC	GCTAGCGGATTTCTTAGGTGC
	3: MH06g0586500	ACACTCTTTCCCTACACGACGGAGGAATAGTGGTACTCTCTCTGTC	GACTGGAGTTCAGACGTGTGCCGTAACAATGTGTCAATGTCTGC	GTACAACACTCTTTCCCTACACGAC	ACAGCTTCTCGTTGGGTGAT
	4: MH09g0378300	ACACTCTTTCCCTACACGACGTATGTGGAAAAGTTTGTACTAACG	GACTGGAGTTCAGACGTGTGCCACTTGATCCCCAGGAAGTAGG	GTACAACACTCTTTCCCTACACGAC	TCAGCTGTCTCCTCCGATCT
	5: MH09g0431500	ACACTCTTTCCCTACACGACGCTTTTGGCTAGTTCTATAAACAGG	GACTGGAGTTCAGACGTGTGCTTTGCGGTCAAGAACTAAAGATTA	GTACAACACTCTTTCCCTACACGAC	AGGGACGAAGGGAGTAAGAGA

Supplementary Table 5. Primer sequences used for enhancer validation in this study (continued).

Distal enhancer	1: chr12:101889-102650	ACACTCTTTCCCTACACGACGATTCTTGCATTTCCCATATTCATAT	GACTGGAGTTCAGACGTGTGCATAATTCTTAGTATTGTTACCTGCC	GTACAACACTCTTTCCCTACACGAC	TGCATTTGGCCCTAGCTACC
	2: chr3:11511997-11512850	ACACTCTTTCCCTACACGACGACTAAACACAGCCTAATACACTCGT	GACTGGAGTTCAGACGTGTGCATCCGAACTGGTCTTTGCGTAG	GTACAACACTCTTTCCCTACACGAC	CTTTGACCCCTCGGCATCTT
	3: chr10:16186041-16186708	ACACTCTTTCCCTACACGACGAAAATACATAGACCTTGACATCACG	GACTGGAGTTCAGACGTGTGCACTCCAAGACGCAGTGTGTATGAC	GTACAACACTCTTTCCCTACACGAC	ACGATTGTACGTGGCTATCTCG
	4: chr1:21627864-21628585	ACACTCTTTCCCTACACGACGTGTAGCAAACCATGGAACCACC	GACTGGAGTTCAGACGTGTGCGAACTCAGCCTTAATCTTGCCTGT	GTACAACACTCTTTCCCTACACGAC	GATCCAACCGGACAAGTGCT
	5: chr7:22561688-22562357	ACACTCTTTCCCTACACGACGGAACTAAACACGGTGCAACCAT	GACTGGAGTTCAGACGTGTGCAATAGAACAAATTAAACTGAAAATAGC	GTACAACACTCTTTCCCTACACGAC	CGCTTGTCTTTTAGCCGGGA
Control	chr2:28412151-28413151	ACACTCTTTCCCTACACGACGTGCATAGGCACAACCATCCC	GACTGGAGTTCAGACGTGTGCAGGACCGGTTCACGAGTTTC	GTACAACACTCTTTCCCTACACGAC	CGGTTATGGGCGGGTCTAAC
