# Supplementary material for: Integrative analysis of reference epigenomes in 20 rice varieties
Source: Nat Commun. 2020 May 27;11:2658. doi: 10.1038/s41467-020-16457-5 (PMC7253419; doi:10.1038/s41467-020-16457-5)
Supplement: Supplementary file 2 — Reporting Summary [file 41467_2020_16457_MOESM2_ESM.pdf]

nature research

Corresponding author(s): Guoliang Li & Xingwang Li

Reporting Summary

Nature Research wishes to improve the reproducibility of the work that we publish. This form provides structure for consistency and transparency in reporting. For further information on Nature Research policies, see Authors & Referees and the Editorial Policy Checklist.

Statistical parameters

When statistical analyses are reported, confirm that the following items are present in the relevant location (e.g. figure legend, table legend, main text, or Methods section).

n/a	Confirmed	
☐	☒	The exact sample size (n) for each experimental group/condition, given as a discrete number and unit of measurement
☐	☒	An indication of whether measurements were taken from distinct samples or whether the same sample was measured repeatedly
☐	☒	The statistical test(s) used AND whether they are one- or two-sided<br>*Only common tests should be described solely by name; describe more complex techniques in the Methods section.*
☒	☐	A description of all covariates tested
☒	☐	A description of any assumptions or corrections, such as tests of normality and adjustment for multiple comparisons
☐	☒	A full description of the statistics including central tendency (e.g. means) or other basic estimates (e.g. regression coefficient) AND variation (e.g. standard deviation) or associated estimates of uncertainty (e.g. confidence intervals)
☐	☒	For null hypothesis testing, the test statistic (e.g. F, t, r) with confidence intervals, effect sizes, degrees of freedom and P value noted<br>*Give P values as exact values whenever suitable.*
☒	☐	For Bayesian analysis, information on the choice of priors and Markov chain Monte Carlo settings
☒	☐	For hierarchical and complex designs, identification of the appropriate level for tests and full reporting of outcomes
☐	☒	Estimates of effect sizes (e.g. Cohen's d, Pearson's r), indicating how they were calculated
☐	☒	Clearly defined error bars<br>*State explicitly what error bars represent (e.g. SD, SE, CI)*

Our web collection on statistics for biologists may be useful.

Software and code

Policy information about availability of computer code

Data collection	All sequencing libraries were prepared in house and raw reads were generated on Illumina high-throughtput sequencing paltform with manufacture's instruction. No software was used for data collection.
Data analysis	Software used include: BWA (0.7.13), Tophat2 (2.1.0), Trimmomatic (0.36),MACS2 (2.1.0), deepTools (2.5.3), BatMeth2, ChromHMM v1.12, BEDTools (2.25.0), Cufflinks (2.2.1), SAMTools (1.3.1), as well as R version 3.2.1 (2015-06-18) and Python to run many of the mentioned programs. Detailed parameters of each of the programs are mentioned in relevant sections in Methods.

For manuscripts utilizing custom algorithms or software that are central to the research but not yet described in published literature, software must be made available to editors/reviewers upon request. We strongly encourage code deposition in a community repository (e.g. GitHub). See the Nature Research guidelines for submitting code & software for further information.

Data

Policy information about availability of data

All manuscripts must include a data availability statement. This statement should provide the following information, where applicable:

- Accession codes, unique identifiers, or web links for publicly available datasets
- A list of figures that have associated raw data
- A description of any restrictions on data availability

All of raw data has been uploaded to the NCBI GEO under accession numbers GSE142570 (https://www.ncbi.nlm.nih.gov/geo/query/acc.cgi?acc=GSE142570)

Field-specific reporting

Please select the best fit for your research. If you are not sure, read the appropriate sections before making your selection.

☒ Life sciences ☐ Behavioural & social sciences ☐ Ecological, evolutionary & environmental sciences

For a reference copy of the document with all sections, see nature.com/authors/policies/ReportingSummary-flat.pdf

Life sciences study design

All studies must disclose on these points even when the disclosure is negative.

Sample size	The effective sample size is the the number of loci studied, which are described through the manuscript.
Data exclusions	No exclusion of data was made.
Replication	All experimental data was reliably reproduced in two independent experiments as indicated in the figure legends.
Randomization	The plants were grown in the same conditions (see Methods) and randomly allocated into experimental groups.
Blinding	No blinding was used since measurements were not vulnerable to observer bias. Whenever separate groups were compared, the same analysis pipelines were performed in parallel.

Reporting for specific materials, systems and methods

Materials & experimental systems

n/a	Involved in the study	
☒	☐	Unique biological materials
☐	☒	Antibodies
☒	☐	Eukaryotic cell lines
☒	☐	Palaeontology
☒	☐	Animals and other organisms
☒	☐	Human research participants

Methods

n/a	Involved in the study	
☐	☒	ChIP-seq
☒	☐	Flow cytometry
☒	☐	MRI-based neuroimaging

Antibodies

Antibodies used	H3K4me1 polyclonal antibody (ABclonal, A2355), H3K4me3 polyclonal antibody (ABclonal, A2357), H3K9me2 monoclonal antibody (Abcam, ab1220), H3K27me3 polyclonal antibody (ABclonal, A2363), H3K27ac polyclonal antibody (ABclonal, A7253), and RNAPII monoclonal antibody (BioLegend, 920102)
Validation	The antibodies have been validated both by the companies and by ourselves. The companies have used dot plot, western blot, immunofluorescence, ChIP-qPCR or ChIP-seq experiments. Please refer to the information below: H3K4me1 antibody: https://www.abclonal.com.cn/catalog/A2355; H3K4me3 antibody: https://www.abclonal.com.cn/catalog/A2357; H3K9me2 antibody: https://www.abcam.cn/histone-h3-di-methyl-k9-antibody-mabcam-1220-chip-grade-ab1220.html; H3K27me3 antibody: https://www.abclonal.com.cn/catalog/A2363; H3K27ac antibody: https://www.abclonal.com.cn/catalog/A7253; These commercial antibodies are well used and reported in many previous publications of our and other labs. We have carefully checked the specificity reports of the antibodies from the companies, and have validated the specificity of the antibodies in house. According to the human ENCODE guidelines, we carried out the primary characterization (western blot) and the secondary characterization

(dot blot) to ensure the specificity of antibodies. ChIP-seq method was also used to test the the specificity of antibodies.

ChIP-seq

Data deposition

☒ Confirm that both raw and final processed data have been deposited in a public database such as GEO.

☒ Confirm that you have deposited or provided access to graph files (e.g. BED files) for the called peaks.

Data access links<br>*May remain private before publication.*	The raw sequence data are available at NCBI GEO under accession numbers GSE142570 (https://www.ncbi.nlm.nih.gov/geo/query/acc.cgi?acc=GSE142570)
Files in database submission	510 data files including 352 ChIP-Seq datasets , 58 RNA-Seq data,58 FAIRE-Seq data, 38 Bisulfite-Seq data and 4 ChIP-reChIP data (summarized in Extended Data Table 2) .<br>MH63_young_leaf_H3K4me3_rep1<br>MH63_young_leaf_H3K4me3_rep2<br>MH63_young_leaf_H3K27ac_rep1<br>MH63_young_leaf_H3K27ac_rep2<br>MH63_young_leaf_H3K27me3_rep1<br>MH63_young_leaf_H3K27me3_rep2<br>MH63_young_leaf_H3K4me1_rep1<br>MH63_young_leaf_H3K4me1_rep2<br>MH63_young_leaf_H3K9me2_rep1<br>MH63_young_leaf_H3K9me2_rep2<br>MH63_young_leaf_RNAPII_rep1<br>MH63_young_leaf_RNAPII_rep2<br>MH63_panicle_H3K4me3_rep1<br>MH63_panicle_H3K4me3_rep2<br>MH63_panicle_H3K27ac_rep1<br>MH63_panicle_H3K27ac_rep2<br>MH63_panicle_H3K27me3_rep1<br>MH63_panicle_H3K27me3_rep2<br>MH63_panicle_H3K4me1_rep1<br>MH63_panicle_H3K4me1_rep2<br>MH63_panicle_H3K9me2_rep1<br>MH63_panicle_H3K9me2_rep2<br>MH63_panicle_RNAPII_rep1<br>MH63_panicle_RNAPII_rep2<br>MH63_root_H3K4me3_rep1<br>MH63_root_H3K4me3_rep2<br>MH63_root_H3K27ac_rep1<br>MH63_root_H3K27ac_rep2<br>MH63_root_H3K27me3_rep1<br>MH63_root_H3K27me3_rep2<br>MH63_root_H3K4me1_rep1<br>MH63_root_H3K4me1_rep2<br>MH63_root_H3K9me2_rep1<br>MH63_root_H3K9me2_rep2<br>MH63_mature_leaf_H3K4me3_rep1<br>MH63_mature_leaf_H3K4me3_rep2<br>MH63_mature_leaf_H3K27ac_rep1<br>MH63_mature_leaf_H3K27ac_rep2<br>MH63_mature_leaf_H3K27me3_rep1<br>MH63_mature_leaf_H3K27me3_rep2<br>MH63_mature_leaf_H3K4me1_rep1<br>MH63_mature_leaf_H3K4me1_rep2<br>MH63_mature_leaf_H3K9me2_rep1<br>MH63_mature_leaf_H3K9me2_rep2<br>MH63_mature_leaf_RNAPII_rep1<br>MH63_mature_leaf_RNAPII_rep2<br>ZS97_young_leaf_H3K4me3_rep1<br>ZS97_young_leaf_H3K4me3_rep2<br>ZS97_young_leaf_H3K27ac_rep1<br>ZS97_young_leaf_H3K27ac_rep2<br>ZS97_young_leaf_H3K27me3_rep1<br>ZS97_young_leaf_H3K27me3_rep2<br>ZS97_young_leaf_H3K4me1_rep1<br>ZS97_young_leaf_H3K4me1_rep2<br>ZS97_young_leaf_H3K9me2_rep1<br>ZS97_young_leaf_H3K9me2_rep2<br>ZS97_young_leaf_RNAPII_rep1<br>ZS97_young_leaf_RNAPII_rep2<br>ZS97_panicle_H3K4me3_rep1

ZS97_panicle_H3K4me3_rep2
ZS97_panicle_H3K27ac_rep1
ZS97_panicle_H3K27ac_rep2
ZS97_panicle_H3K27me3_rep1
ZS97_panicle_H3K27me3_rep2
ZS97_panicle_H3K4me1_rep1
ZS97_panicle_H3K4me1_rep2
ZS97_panicle_H3K9me2_rep1
ZS97_panicle_H3K9me2_rep2
ZS97_panicle_RNAPII_rep1
ZS97_panicle_RNAPII_rep2
ZS97_root_H3K4me3_rep1
ZS97_root_H3K4me3_rep2
ZS97_root_H3K27ac_rep1
ZS97_root_H3K27ac_rep2
ZS97_root_H3K27me3_rep1
ZS97_root_H3K27me3_rep2
ZS97_root_H3K4me1_rep1
ZS97_root_H3K4me1_rep2
ZS97_root_H3K9me2_rep1
ZS97_root_H3K9me2_rep2
ZS97_mature_leaf_H3K4me3_rep1
ZS97_mature_leaf_H3K4me3_rep2
ZS97_mature_leaf_H3K27ac_rep1
ZS97_mature_leaf_H3K27ac_rep2
ZS97_mature_leaf_H3K27me3_rep1
ZS97_mature_leaf_H3K27me3_rep2
ZS97_mature_leaf_H3K4me1_rep1
ZS97_mature_leaf_H3K4me1_rep2
ZS97_mature_leaf_H3K9me2_rep1
ZS97_mature_leaf_H3K9me2_rep2
ZS97_mature_leaf_RNAPII_rep1
ZS97_mature_leaf_RNAPII_rep2
Nip_young_leaf_H3K4me3_1
Nip_young_leaf_H3K4me3_2
Nip_young_leaf_H3K27ac_1
Nip_young_leaf_H3K27ac_2
Nip_young_leaf_H3K27me3_1
Nip_young_leaf_H3K27me3_2
Nip_young_leaf_H3K4me1_1
Nip_young_leaf_H3K4me1_2
Nip_young_leaf_H3K9me2_1
Nip_young_leaf_H3K9me2_2
Nip_young_leaf_RNAPII_1
Nip_young_leaf_RNAPII_2
Nip_panicle_H3K4me3_1
Nip_panicle_H3K4me3_2
Nip_panicle_H3K27ac_1
Nip_panicle_H3K27ac_2
Nip_panicle_H3K27me3_1
Nip_panicle_H3K27me3_2
Nip_panicle_H3K4me1_1
Nip_panicle_H3K4me1_2
Nip_panicle_H3K9me2_1
Nip_panicle_H3K9me2_2
Nip_panicle_RNAPII_1
Nip_panicle_RNAPII_2
Nip_root_H3K4me3_rep1
Nip_root_H3K4me3_rep2
Nip_root_H3K27ac_rep1
Nip_root_H3K27ac_rep2
Nip_root_H3K27me3_rep1
Nip_root_H3K27me3_rep2
Nip_root_H3K4me1_rep1
Nip_root_H3K4me1_rep2
Nip_root_H3K9me2_rep1
Nip_root_H3K9me2_rep2
Nip_mature_leaf_H3K4me3_rep1
Nip_mature_leaf_H3K4me3_rep2
Nip_mature_leaf_H3K27ac_rep1
Nip_mature_leaf_H3K27ac_rep2
Nip_mature_leaf_H3K27me3_rep1
Nip_mature_leaf_H3K27me3_rep2
Nip_mature_leaf_H3K4me1_rep1
Nip_mature_leaf_H3K4me1_rep2

Nip_mature_leaf_H3K9me2_rep1
Nip_mature_leaf_H3K9me2_rep2
Nip_mature_leaf_RNAPII_rep1
Nip_mature_leaf_RNAPII_rep2
MH63_H3K9me2_5g_standard
MH63_H3K9me2_0.01g_2
MH63_H3K9me2_0.05g_2
MH63_H3K9me2_0.2g_2
ZS97_H3K4me3_5g_standard
ZS97_H3K4me3_0.01g_1
ZS97_H3K4me3_0.01g_2
ZS97_H3K4me3_0.05g_1
ZS97_H3K4me3_0.05g_2
ZS97_H3K4me3_0.2g_2
W105_young_leaf_H3K4me3_rep1
W105_young_leaf_H3K4me3_rep2
W105_young_leaf_H3K27ac_rep1
W105_young_leaf_H3K27ac_rep2
W105_young_leaf_H3K27me3_rep1
W105_young_leaf_H3K27me3_rep2
W105_young_leaf_H3K4me1_rep1
W105_young_leaf_H3K4me1_rep2
W105_young_leaf_H3K9me2_rep1
W105_young_leaf_H3K9me2_rep2
W105_young_leaf_RNAPII_rep1
W105_young_leaf_RNAPII_rep2
W286_young_leaf_H3K4me3_rep1
W286_young_leaf_H3K4me3_rep2
W286_young_leaf_H3K27ac_rep1
W286_young_leaf_H3K27ac_rep2
W286_young_leaf_H3K27me3_rep1
W286_young_leaf_H3K27me3_rep2
W286_young_leaf_H3K4me1_rep1
W286_young_leaf_H3K4me1_rep2
W286_young_leaf_H3K9me2_rep1
W286_young_leaf_H3K9me2_rep2
W286_young_leaf_RNAPII_rep1
W286_young_leaf_RNAPII_rep2
C019_young_leaf_H3K4me3_rep1
C019_young_leaf_H3K4me3_rep2
C019_young_leaf_H3K27ac_rep1
C019_young_leaf_H3K27ac_rep2
C019_young_leaf_H3K27me3_rep1
C019_young_leaf_H3K27me3_rep2
C019_young_leaf_H3K4me1_rep1
C019_young_leaf_H3K4me1_rep2
C019_young_leaf_H3K9me2_rep1
C019_young_leaf_H3K9me2_rep2
C019_young_leaf_RNAPII_rep1
C019_young_leaf_RNAPII_rep2
C135_young_leaf_H3K4me3_rep1
C135_young_leaf_H3K4me3_rep2
C135_young_leaf_H3K27ac_rep1
C135_young_leaf_H3K27ac_rep2
C135_young_leaf_H3K27me3_rep1
C135_young_leaf_H3K27me3_rep2
C135_young_leaf_H3K4me1_rep1
C135_young_leaf_H3K4me1_rep2
C135_young_leaf_H3K9me2_rep1
C135_young_leaf_H3K9me2_rep2
C135_young_leaf_RNAPII_rep1
C135_young_leaf_RNAPII_rep2
C139_young_leaf_H3K4me3_rep1
C139_young_leaf_H3K4me3_rep2
C139_young_leaf_H3K27ac_rep1
C139_young_leaf_H3K27ac_rep2
C139_young_leaf_H3K27me3_rep1
C139_young_leaf_H3K27me3_rep2
C139_young_leaf_H3K4me1_rep1
C139_young_leaf_H3K4me1_rep2
C139_young_leaf_H3K9me2_rep1
C139_young_leaf_H3K9me2_rep2
C139_young_leaf_RNAPII_rep1
C139_young_leaf_RNAPII_rep2
C151_young_leaf_H3K4me3_rep1

C151_young_leaf_H3K4me3_rep2
C151_young_leaf_H3K27ac_rep1
C151_young_leaf_H3K27ac_rep2
C151_young_leaf_H3K27me3_rep1
C151_young_leaf_H3K27me3_rep2
C151_young_leaf_H3K4me1_rep1
C151_young_leaf_H3K4me1_rep2
C151_young_leaf_H3K9me2_rep1
C151_young_leaf_H3K9me2_rep2
C151_young_leaf_RNAPII_rep1
C151_young_leaf_RNAPII_rep2
C148_young_leaf_H3K4me3_rep1
C148_young_leaf_H3K4me3_rep2
C148_young_leaf_H3K27ac_rep1
C148_young_leaf_H3K27ac_rep2
C148_young_leaf_H3K27me3_rep1
C148_young_leaf_H3K27me3_rep2
C148_young_leaf_H3K4me1_rep1
C148_young_leaf_H3K4me1_rep2
C148_young_leaf_H3K9me2_rep1
C148_young_leaf_H3K9me2_rep2
C148_young_leaf_RNAPII_rep1
C148_young_leaf_RNAPII_rep2
W161_young_leaf_H3K4me3_rep1
W161_young_leaf_H3K4me3_rep2
W161_young_leaf_H3K27ac_rep1
W161_young_leaf_H3K27ac_rep2
W161_young_leaf_H3K27me3_rep1
W161_young_leaf_H3K27me3_rep2
W161_young_leaf_H3K4me1_rep1
W161_young_leaf_H3K4me1_rep2
W161_young_leaf_H3K9me2_rep1
W161_young_leaf_H3K9me2_rep2
W161_young_leaf_RNAPII_rep1
W161_young_leaf_RNAPII_rep2
W169_young_leaf_H3K4me3_rep1
W169_young_leaf_H3K4me3_rep2
W169_young_leaf_H3K27ac_rep1
W169_young_leaf_H3K27ac_rep2
W169_young_leaf_H3K27me3_rep1
W169_young_leaf_H3K27me3_rep2
W169_young_leaf_H3K4me1_rep1
W169_young_leaf_H3K4me1_rep2
W169_young_leaf_H3K9me2_rep1
W169_young_leaf_H3K9me2_rep2
W169_young_leaf_RNAPII_rep1
W169_young_leaf_RNAPII_rep2
C051_young_leaf_H3K4me3_rep1
C051_young_leaf_H3K4me3_rep2
C051_young_leaf_H3K27ac_rep1
C051_young_leaf_H3K27ac_rep2
C051_young_leaf_H3K27me3_rep1
C051_young_leaf_H3K27me3_rep2
C051_young_leaf_H3K4me1_rep1
C051_young_leaf_H3K4me1_rep2
C051_young_leaf_H3K9me2_rep1
C051_young_leaf_H3K9me2_rep2
C051_young_leaf_RNAPII_rep1
C051_young_leaf_RNAPII_rep2
W125_young_leaf_H3K4me3_rep1
W125_young_leaf_H3K4me3_rep2
W125_young_leaf_H3K27ac_rep1
W125_young_leaf_H3K27ac_rep2
W125_young_leaf_H3K27me3_rep1
W125_young_leaf_H3K27me3_rep2
W125_young_leaf_H3K4me1_rep1
W125_young_leaf_H3K4me1_rep2
W125_young_leaf_H3K9me2_rep1
W125_young_leaf_H3K9me2_rep2
W125_young_leaf_RNAPII_rep1
W125_young_leaf_RNAPII_rep2
W128_young_leaf_H3K4me3_rep1
W128_young_leaf_H3K4me3_rep2
W128_young_leaf_H3K27ac_rep1
W128_young_leaf_H3K27ac_rep2

W128_young_leaf_H3K27me3_rep1
W128_young_leaf_H3K27me3_rep2
W128_young_leaf_H3K4me1_rep1
W128_young_leaf_H3K4me1_rep2
W128_young_leaf_H3K9me2_rep1
W128_young_leaf_H3K9me2_rep2
W128_young_leaf_RNAPII_rep1
W128_young_leaf_RNAPII_rep2
W261_young_leaf_H3K4me3_rep1
W261_young_leaf_H3K4me3_rep2
W261_young_leaf_H3K27ac_rep1
W261_young_leaf_H3K27ac_rep2
W261_young_leaf_H3K27me3_rep1
W261_young_leaf_H3K27me3_rep2
W261_young_leaf_H3K4me1_rep1
W261_young_leaf_H3K4me1_rep2
W261_young_leaf_H3K9me2_rep1
W261_young_leaf_H3K9me2_rep2
W261_young_leaf_RNAPII_rep1
W261_young_leaf_RNAPII_rep2
W294_young_leaf_H3K4me3_rep1
W294_young_leaf_H3K4me3_rep2
W294_young_leaf_H3K27ac_rep1
W294_young_leaf_H3K27ac_rep2
W294_young_leaf_H3K27me3_rep1
W294_young_leaf_H3K27me3_rep2
W294_young_leaf_H3K4me1_rep1
W294_young_leaf_H3K4me1_rep2
W294_young_leaf_H3K9me2_rep1
W294_young_leaf_H3K9me2_rep2
W294_young_leaf_RNAPII_rep1
W294_young_leaf_RNAPII_rep2
W257_young_leaf_H3K4me3_rep1
W257_young_leaf_H3K4me3_rep2
W257_young_leaf_H3K27ac_rep1
W257_young_leaf_H3K27ac_rep2
W257_young_leaf_H3K27me3_rep1
W257_young_leaf_H3K27me3_rep2
W257_young_leaf_H3K4me1_rep1
W257_young_leaf_H3K4me1_rep2
W257_young_leaf_H3K9me2_rep1
W257_young_leaf_H3K9me2_rep2
W257_young_leaf_RNAPII_rep1
W257_young_leaf_RNAPII_rep2
W081_young_leaf_H3K4me3_rep1
W081_young_leaf_H3K4me3_rep2
W081_young_leaf_H3K27ac_rep1
W081_young_leaf_H3K27ac_rep2
W081_young_leaf_H3K27me3_rep1
W081_young_leaf_H3K27me3_rep2
W081_young_leaf_H3K4me1_rep1
W081_young_leaf_H3K4me1_rep2
W081_young_leaf_H3K9me2_rep1
W081_young_leaf_H3K9me2_rep2
W081_young_leaf_RNAPII_rep1
W081_young_leaf_RNAPII_rep2
W306_young_leaf_H3K4me3_rep1
W306_young_leaf_H3K4me3_rep2
W306_young_leaf_H3K27ac_rep1
W306_young_leaf_H3K27ac_rep2
W306_young_leaf_H3K27me3_rep1
W306_young_leaf_H3K27me3_rep2
W306_young_leaf_H3K4me1_rep1
W306_young_leaf_H3K4me1_rep2
W306_young_leaf_H3K9me2_rep1
W306_young_leaf_H3K9me2_rep2
W306_young_leaf_RNAPII_rep1
W306_young_leaf_RNAPII_rep2
MH63_young_leaf_RNA_rep1
MH63_young_leaf_RNA_rep2
MH63_panicle_RNA_rep1
MH63_panicle_RNA_rep2
MH63_root_RNA_rep1
MH63_root_RNA_rep2
MH63_mature_leaf_RNA_rep1

MH63_mature_leaf_RNA_rep2
ZS_young_leaf_RNA_rep1
ZS_young_leaf_RNA_rep2
ZS_panicle_RNA_rep1
ZS_panicle_RNA_rep2
ZS_root_RNA_rep1
ZS_root_RNA_rep2
ZS97_mature_leaf_RNA_rep1
ZS97_mature_leaf_RNA_rep2
Nip_young_leaf_RNA_rep1
Nip_young_leaf_RNA_rep2
Nip_panicle_RNA_rep1
Nip_panicle_RNA_rep2
Nip_root_RNA_rep1
Nip_root_RNA_rep2
Nip_mature_leaf_RNA_rep1
Nip_mature_leaf_RNA_rep2
W105_young_leaf_RNA_rep1
W105_young_leaf_RNA_rep2
W286_young_leaf_RNA_rep1
W286_young_leaf_RNA_rep2
C019_young_leaf_RNA_rep1
C019_young_leaf_RNA_rep2
C135_young_leaf_RNA_rep1
C135_young_leaf_RNA_rep2
C139_young_leaf_RNA_rep1
C139_young_leaf_RNA_rep2
C151_young_leaf_RNA_rep1
C151_young_leaf_RNA_rep2
C148_young_leaf_RNA_rep1
C148_young_leaf_RNA_rep2
W161_young_leaf_RNA_rep1
W161_young_leaf_RNA_rep2
W169_young_leaf_RNA_rep1
W169_young_leaf_RNA_rep2
C051_young_leaf_RNA_rep1
C051_young_leaf_RNA_rep2
W125_young_leaf_RNA_rep1
W125_young_leaf_RNA_rep2
W128_young_leaf_RNA_rep1
W128_young_leaf_RNA_rep2
W261_young_leaf_RNA_rep1
W261_young_leaf_RNA_rep2
W294_young_leaf_RNA_rep1
W294_young_leaf_RNA_rep2
W257_young_leaf_RNA_rep1
W257_young_leaf_RNA_rep2
W081_young_leaf_RNA_rep1
W081_young_leaf_RNA_rep2
W306_young_leaf_RNA_rep1
W306_young_leaf_RNA_rep2
MH63_young_leaf_FAIRE_rep1
MH63_young_leaf_FAIRE_rep2
MH63_panicle_FAIRE_rep1
MH63_panicle_FAIRE_rep2
MH63_root_FAIRE_rep1
MH63_root_FAIRE_rep2
MH63_mature_leaf_FAIRE_rep1
MH63_mature_leaf_FAIRE_rep2
ZS97_young_leaf_FAIRE_rep1
ZS97_young_leaf_FAIRE_rep2
ZS97_panicle_FAIRE_rep1
ZS97_panicle_FAIRE_rep2
ZS97_root_FAIRE_rep1
ZS97_root_FAIRE_rep2
ZS97_mature_leaf_FAIRE_rep1
ZS97_mature_leaf_FAIRE_rep2
Nip_young_leaf_FAIRE_rep1
Nip_young_leaf_FAIRE_rep2
Nip_panicle_FAIRE_rep1
Nip_panicle_FAIRE_rep2
Nip_root_FAIRE_1
Nip_root_FAIRE_2
Nip_mature_leaf_FAIRE_1
Nip_mature_leaf_FAIRE_2

W105_young_leaf_FAIRE_rep1
W105_young_leaf_FAIRE_rep2
W286_young_leaf_FAIRE_rep1
W286_young_leaf_FAIRE_rep2
C019_young_leaf_FAIRE_rep1
C019_young_leaf_FAIRE_rep2
C135_young_leaf_FAIRE_rep1
C135_young_leaf_FAIRE_rep2
C139_young_leaf_FAIRE_rep1
C139_young_leaf_FAIRE_rep2
C151_young_leaf_FAIRE_rep1
C151_young_leaf_FAIRE_rep2
C148_young_leaf_FAIRE_rep1
C148_young_leaf_FAIRE_rep2
W161_young_leaf_FAIRE_rep1
W161_young_leaf_FAIRE_rep2
W169_young_leaf_FAIRE_rep1
W169_young_leaf_FAIRE_rep2
C051_young_leaf_FAIRE_rep1
C051_young_leaf_FAIRE_rep2
W125_young_leaf_FAIRE_rep1
W125_young_leaf_FAIRE_rep2
W128_young_leaf_FAIRE_rep1
W128_young_leaf_FAIRE_rep2
W261_young_leaf_FAIRE_rep1
W261_young_leaf_FAIRE_rep2
W294_young_leaf_FAIRE_rep1
W294_young_leaf_FAIRE_rep2
W257_young_leaf_FAIRE_rep1
W257_young_leaf_FAIRE_rep2
W081_young_leaf_FAIRE_rep1
W081_young_leaf_FAIRE_rep2
W306_young_leaf_FAIRE_rep1
W306_young_leaf_FAIRE_rep2
MH63_young_leaf_DNAmeth_1
MH63_young_leaf_DNAmeth_P1 additional
MH63_panicle_DNAmeth_1
MH63_panicle_DNAmeth_P1 additional
MH63_root_DNAmethy_1
MH63_root_DNAmethy_P1 additional
MH63_matrue_leaf_DNAmeth_1
MH63_matrue_leaf_DNAmeth_P1 additional
ZS97_young_leaf_DNAmeth_1
ZS97_young_leaf_DNAmeth_P1 additional
ZS97_panicle_DNAmeth_1
ZS97_panicle_DNAmeth_P1 additional
ZS97_panicle_DNAmeth_P2 additional
ZS97_panicle_DNAmeth_P3 additional
ZS97_root_DNAmeth_1
ZS97_root_DNAmeth_P1 additional
Nip_young_leaf_DNAmeth_1
Nip_panicle_DNAmeth_1
Nip_root_DNAmeth
Nip_mature_leaf_DNAmeth
ZS97_mature_leaf_DNAmeth
W105_young_leaf_DNAmeth
W286_young_leaf_DNAmeth
C019_young_leaf_DNAmeth
C135_young_leaf_DNAmeth
C139_young_leaf_DNAmeth
C151_young_leaf_DNAmeth
C148_young_leaf_DNAmeth
W161_young_leaf_DNAmeth
W169_young_leaf_DNAmeth
C051_young_leaf_DNAmeth
W125_young_leaf_DNAmeth
W128_young_leaf_DNAmeth
W261_young_leaf_DNAmeth
W294_young_leaf_DNAmeth
W257_young_leaf_DNAmeth
W081_young_leaf_DNAmeth
W306_young_leaf_DNAmeth
MH63_young_leaf_H3K4me3_H3K27ac
MH63_young_leaf_H3K27ac_H3K4me3
MH63_young_leaf_H3K9me2_H3K4me1

MH63_young_leaf_H3K4me1_H3K9me2

Genome browser session (e.g. UCSC)

Please use the following link: http://218.199.68.190:8008/basic/main/MH63/ with username: "rice_encode" and password: "rice_encode" to visualize peak files.

Methodology

Replicates

Two biological replicates for each histone mark (H3K4me3, H3K4me1,H3K27ac,H3K27me3,H3K9me2), RNA-Seq and FAIRE-Seq in examined tissues of 3 varieties and another 17 varieties in young leaf.

Sequencing depth

About 13 million pair-end (2x150bp) raw reads on average for each experiment.

Antibodies

H3K4me1 polyclonal antibody (ABclonal, A2355), H3K4me3 polyclonal antibody (ABclonal, A2357), H3K9me2 monoclonal antibody (Abcam, ab1220), H3K27me3 polyclonal antibody (ABclonal, A2363), H3K27ac polyclonal antibody (ABclonal, A7253), and RNAPII monoclonal antibody (BioLegend, 920102)

Peak calling parameters

For narrow peak calling: macs2 callpeak function with "callpeak –t <input file> -c <control file> -f BAM –n <output peak file > -B -g 3.6e+8".For broad peak calling: broad-peak mode was used in MACS2 with FDR < 0.1, see Methods section in the manuscript for the details

Data quality

We used FRiP (fraction of reads in peaks), NSC (normalized strand coefficient) and RSC (relative strand correlation) to evaluate our data quality following Human ENCODE project guidelines. We also visualized peak signals on genome browser for each dataset. The number of narrow peak (FDR < 0.05, default parameter) is from 12,000 to 28,000; the number of broad peak (FDR < 0.1, default parameter) is from 11,000 to 17,000.

Software

BWA (0.7.13), Tophat2 (2.1.0), Trimmomatic (0.36),MACS2 (2.1.0), deepTools (2.5.3), BatMeth2, ChromHMM v1.12, BEDTools (2.25.0), Cufflinks (2.2.1), SAMTools (1.3.1),
